# Childhood obesity interventions among Latinos residing in the US–Mexico border: a systematic review and meta-analysis of obesity outcomes

**DOI:** 10.1093/abm/kaag056

**Published:** 2026-09-16

**Authors:** Patricia Dionicio, Zahid Garcia Lopez, Kimberly D Sánchez, Bianca Vargas Tequida, Alva Nidia Laprada Villegas, Juan Pablo Paullada, Daniela Guadalupe Gonzalez-Valencia, María Isabel Ortega, Siddarth Singh, Noe C Crespo

**Affiliations:** School of Public Health, University of California San Diego/San Diego State University Joint Doctoral Program in Public Health, San Diego, CA 92182, United States; Binational Alliance for Transborder Community Health, San Diego State University, Universidad Autónoma de Baja California Mexicali, Centro de Investigación en Alimentación y Desarrollo, Baja California, Mexicali 21280, Mexico; Binational Alliance for Transborder Community Health, San Diego State University, Universidad Autónoma de Baja California Mexicali, Centro de Investigación en Alimentación y Desarrollo, Baja California, Mexicali 21280, Mexico; Departamento de Nutrición Pública y Salud, Centro de Investigación en Alimentación y Desarrollo, A.C., Hermosillo, Sonora 83304, Mexico; Institute for Behavioral and Community Health (IBACH), San Diego State University, San Diego, CA 92123, United States; Binational Alliance for Transborder Community Health, San Diego State University, Universidad Autónoma de Baja California Mexicali, Centro de Investigación en Alimentación y Desarrollo, Baja California, Mexicali 21280, Mexico; Health Sciences Research Institute, University of California Merced, Merced, CA 95343, United States; Binational Alliance for Transborder Community Health, San Diego State University, Universidad Autónoma de Baja California Mexicali, Centro de Investigación en Alimentación y Desarrollo, Baja California, Mexicali 21280, Mexico; Institute for Behavioral and Community Health (IBACH), San Diego State University, San Diego, CA 92123, United States; Binational Alliance for Transborder Community Health, San Diego State University, Universidad Autónoma de Baja California Mexicali, Centro de Investigación en Alimentación y Desarrollo, Baja California, Mexicali 21280, Mexico; Facultad de Medicina y Nutrición, Universidad Autónoma de Baja California Mexicali, Baja California, Mexicali 21000, Mexico; Facultad de Medicina y Nutrición, Universidad Autónoma de Baja California Mexicali, Baja California, Mexicali 21000, Mexico; Binational Alliance for Transborder Community Health, San Diego State University, Universidad Autónoma de Baja California Mexicali, Centro de Investigación en Alimentación y Desarrollo, Baja California, Mexicali 21280, Mexico; Facultad de Medicina y Nutrición, Universidad Autónoma de Baja California Mexicali, Baja California, Mexicali 21000, Mexico; Binational Alliance for Transborder Community Health, San Diego State University, Universidad Autónoma de Baja California Mexicali, Centro de Investigación en Alimentación y Desarrollo, Baja California, Mexicali 21280, Mexico; Departamento de Nutrición Pública y Salud, Centro de Investigación en Alimentación y Desarrollo, A.C., Hermosillo, Sonora 83304, Mexico; Division of Gastroenterology and Hepatology, Department of Medicine, Mayo Clinic Arizona, Scottsdale, AZ 85259, United States; Binational Alliance for Transborder Community Health, San Diego State University, Universidad Autónoma de Baja California Mexicali, Centro de Investigación en Alimentación y Desarrollo, Baja California, Mexicali 21280, Mexico; Institute for Behavioral and Community Health (IBACH), San Diego State University, San Diego, CA 92123, United States; Facultad de Medicina y Nutrición, Universidad Autónoma de Baja California Mexicali, Baja California, Mexicali 21000, Mexico; School of Public Health, San Diego State University, San Diego, CA 92182, United States

**Keywords:** childhood obesity, pediatric, Hispanic youth, bi-national health, border region, meta-analysis

## Abstract

**Objective:**

The United States and Mexico have similarly high childhood obesity trends, especially among underserved populations and communities living in the US–Mexico border region. To date, limited research has compared childhood obesity prevention interventions in both US and Mexico border states. This systematic literature review and meta-analysis aimed to evaluate the efficacy of childhood obesity interventions on obesity outcomes among Hispanic/Latino children residing in US–Mexico border states.

**Methods:**

A systematic literature review was conducted using multiple US and Mexico-based databases through February 2026. The search focused on prospective randomized controlled trials or quasi-experimental studies examining the effect of behavioral interventions on preventing childhood obesity in children aged 3-12 years residing in the US or Mexico border states. Random-effects models were applied using the inverse-variance weight method to assess intervention effects on body mass index (BMI), BMI *z*-scores, BMI percentile, waist circumference, and body fat percentage. Subgroup and meta-regression analyses were conducted to assess moderators of intervention efficacy based on parental involvement, country, border region, intervention target level, and intervention duration.

**Results:**

A total of 7,759 studies were initially identified, and 90 studies met the inclusion criteria for full extraction and analysis. Of the 90 included studies, 79% were conducted in US border states and 60% were quasi experimental studies. Meta-analyses demonstrated no significant improvements in obesity outcomes across the studies. In quasi-experimental studies with a control group, interventions that included parental involvement demonstrated no significant changes in BMI *z*-scores (*d* = −0.01; 95% CI, −0.12 to 1.26), whereas interventions without parental involvement demonstrated moderate increases in BMI *z*-scores (*d = *0.67; 95% CI, 0.03 to 1.31).

**Conclusions:**

Childhood obesity prevention interventions conducted in US–Mexico border states or within the border region were not associated with improvements in obesity outcomes, regardless of measure or study design. However, parental involvement may play a role in preventing substantial increases in BMI. Future research should examine intervention effects on obesity-related biomarkers, physical fitness, and dietary outcomes, which may provide more sensitive indicators of efficacy.

**Clinical trial registration number:**

International Platform of Registered Systematic Review and Meta-analysis Protocols (INPLASY202440046, doi: 10.37766/inplasy2024.4.0046).

## Introduction

The United States (US) and Mexico have similarly high childhood obesity trends, especially among underserved populations and communities living in the US–Mexico border region. In the US, Latino children have the highest prevalence of obesity (26%) compared to non-Hispanic Black (25%), non-Hispanic White (17%), and non-Hispanic Asian (9.0%) children.^1^ In Mexico, childhood obesity rates (15%) are among the highest in Latin America compared to Costa Rica (12.3%), El Salvador (11.7%), and Colombia (7.0%).^2^ Of particular concern, obesity rates are greater among Latino children living in border states such as California (18.7%), Arizona (14.4%), New Mexico (12.7%), and Texas (17.6%) compared to non-Hispanic White children of the same age (obesity rates range between 10.5% and 11.7%).^3^ In Mexico, the greatest obesity rates have been observed in northern states bordering the United States including Baja California, Sonora, Chihuahua, Coahuila, Nuevo León, and Tamaulipas compared to the national average (10.1% vs. 8.1%, respectively).^4^ These data demonstrate a clear need to address obesity in both Mexico and the US, particularly among communities living in the US–Mexico border region.

The US–Mexico border region is defined as 62 miles (100 kilometers) north and south of the border and consists of 4 US states (ie, Arizona, California, New Mexico, and Texas) and 6 Mexican states (ie, Baja California Norte, Chihuahua, Coahuila, Nuevo León, Sonora, and Tamaulipas). Latino children who reside in US border regions experience a unique socio-cultural environment that may increase their risk for obesity. For example, several US counties across the US–Mexico border have poverty rates 2.5 times higher than the national average.^5^ Households with a high poverty level are more likely to experience food insecurity, purchase calorie-dense food, and be physically inactive; all of which are risk factors for childhood obesity.^6^^,^^7^

It is well documented that health behaviors (eg, physical activity and diet), social factors (eg, family and community involvement), environmental factors (eg, food and built environment, walkability), and policies (eg, sugar-sweetened beverage tax) all contribute to obesity in both the US and Mexico.^8^^,^^9^ Among children, family-based approaches such as parent involvement and interventions of longer duration can result in greater effects on obesity outcomes.^8^ Previous obesity prevention reviews indicate that multi-level and theory-driven approaches result in greater improvements in child body mass index (BMI) and waist circumference.^10^ However, there are currently no known systematic literature reviews or meta-analyses that have assessed the effects of behavioral interventions on obesity outcomes among Latino children residing in US–Mexico border states or border regions. Such data are critical to identify effective strategies tailored to these unique socio-cultural and environmental contexts. The present systematic review and meta-analysis evaluated the efficacy of childhood obesity prevention interventions with behavior change approaches (eg, physical activity and/or diet) on obesity outcomes among children living in US–Mexico border states. Potential moderating effects by duration, parent involvement, country, border region, and intervention target level were also explored.

## Methods

### Study design

This study followed the preferred reporting items for systematic reviews and meta-analyses (PRISMA) guidelines^11^ to conduct a systematic literature review and meta-analysis on childhood obesity interventions in US–Mexico border states. The protocol was registered in the International Platform of Registered Systematic Review and Meta-analysis Protocols (INPLASY202440046). Protocol registration occurred after conducting the literature search but prior to analysis.

### Search strategy

An exhaustive search was conducted initially in August 2022 and updated in February 2026 for peer-reviewed articles and grey literature published through February 3, 2026. Electronic records and databases included MEDLINE/PubMed, CINAHL, American Psychological Association PsycINFO, Cochrane Library, Scopus, Latin American & Caribbean Health Sciences Literature (LILACS), EBSCOHost, Web of Science, Sport Discus, Embase, Global Health, Bibliomap, DoPHER, and Virtual Health Library. For US grey literature, the following search engines and databases were searched: Google Scholar, Sociological Abstracts, ProQuest: Theses and Dissertations, Social Science Research Network, Preventing Chronic Disease, American Public Health Association conference abstracts, and Worldcat. For search engines with large quantities of results and limited options to adjust search parameters (ie, Google Scholar), only the first 10 pages of results were reviewed. In addition to these sources, the US grey literature review included hand searching for relevant programs and initiatives from local and state organizations websites in border regions (eg, San Diego County Department of Public Health website). For Mexico, the following grey literature sources were searched for theses, dissertations, and unpublished manuscripts: Repositorio Institucional Unison, Universidad de Sonora, Centro de Investigación en Alimentación y Desarrollo A.C., Universidad de Navojoa, Universidad Autónoma de Nuevo León, Universidad Autónoma de Ciudad Juárez, Universidad de Montemorelos, Mexican Association of Members of Faculties and Schools of Nutrition A.C. abstracts, Congreso del Colegio Mexicano de Nutriólogos, and Universidad Autónoma de México repository. Keywords were based on medical subject headings defined *a priori.* A detailed list of keywords and search terms can be located in Material S1. Briefly, keywords related to “obesity,” “pre-school and school age children,” “Hispanic/Latino,” “physical activity,” “sedentary behavior,” “diet and nutrition,” and “intervention” were entered into databases and search engines.

**Figure 1:**
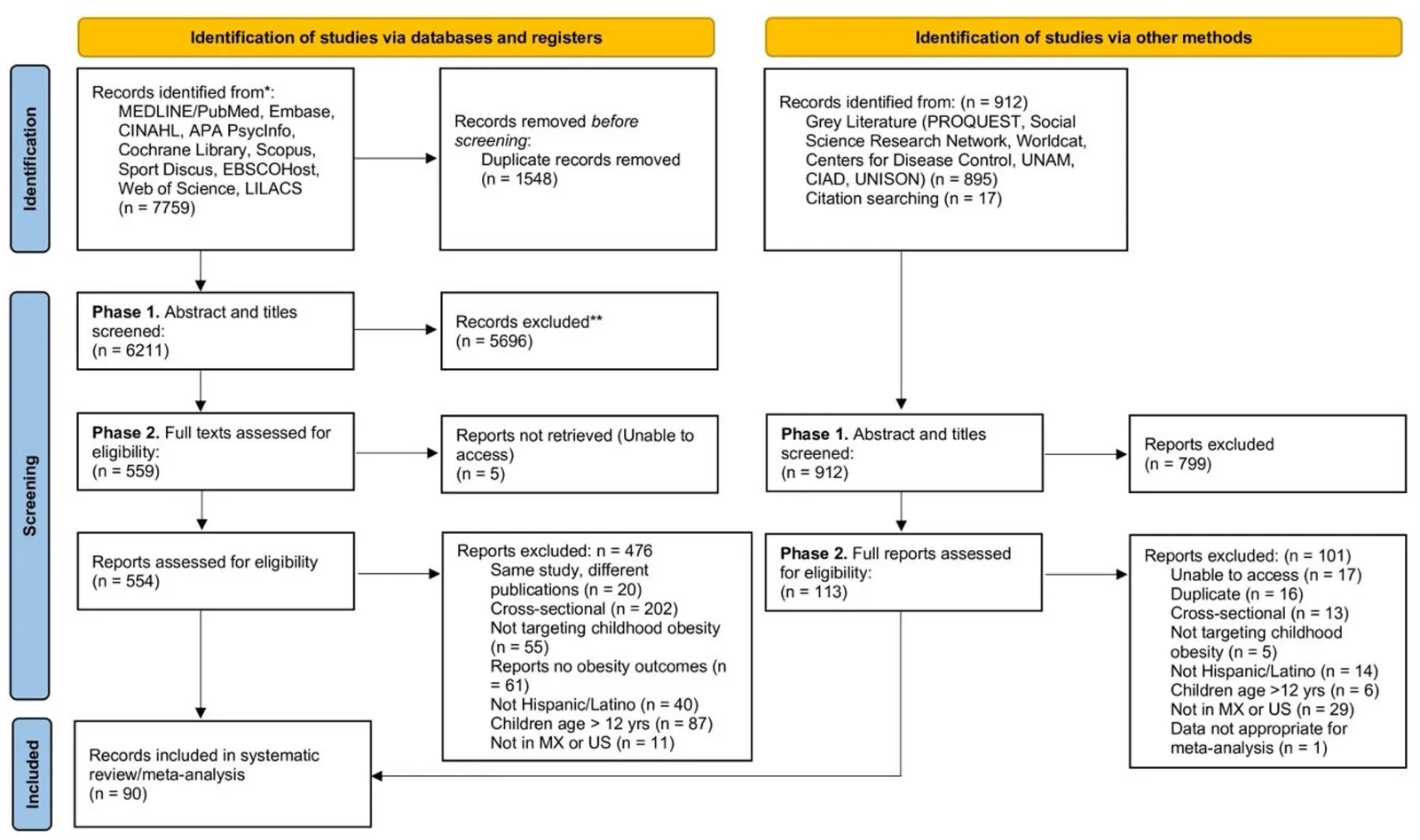
Meta-analysis and systematic review PRISMA diagram. Abbreviations: MX, Mexico; US, United States.

Articles were reviewed in a 3-phase process (PRISMA diagram in Figure 1): phase 1 (abstract review), phase 2 (full text review), and phase 3 (data extraction). During phase 1, abstracts were reviewed and screened for meeting inclusion and exclusion criteria. In phase 2, two researchers reviewed the full-text article, and a third reviewer was responsible for resolving discrepancies. In Phase 3, study data were extracted by two independent reviewers. The grey literature was reviewed using the same procedures as for articles published in scientific journals.

### Study selection

Inclusion criteria for studies were: (1) children of preschool and school age (ie, 3 to 12 years), (2) children were overweight and/or at-risk for obesity or already classified as obese, (3) study conducted in border states of Mexico or the US, (4) participants were primarily Hispanic/Latino (ie, greater than 50%), (5) written in English or Spanish, (6) randomized controlled trial or quasi-experimental study, and (7) intervention strategies targeted physical activity or fitness, sedentary behavior, or dietary behaviors. Exclusion criteria were studies that used a prescribed diet or medication to reduce weight, studies conducted among clinical samples (eg, children with cancer), and studies with a cross-sectional or observational study design.

### Data extraction

The extracted data were organized in Excel and included several study characteristics such as authors, year published, study design, sample sizes for both intervention and control groups, child age, percentage of female participants, percentage of Hispanic/Latino participants, and parent involvement in the intervention. Details about each intervention were also gathered, such as setting (ie, city, state, and country), intervention name, duration, frequency, theoretical framework, behavioral focus (ie, nutrition, physical activity, or sedentary behavior), modality of delivery (eg, teachers, health educators, community health workers), intervention target level (ie, individual, interpersonal, community, policy, multi-level), and descriptions of both intervention and control groups. Lastly, specific results were extracted for each obesity indicator, including the statistical analyses used, raw data to calculate effect sizes (ie, sample size, mean, SD or variance), and effect size when available. In instances where insufficient data were reported to compute effect sizes, an email requesting missing data was sent to corresponding authors.

### Quality assessment

The Cochrane recommended Risk of Bias 2 (RoB2) tool was used to assess the quality of the included randomized controlled trials and quasi-experimental studies with a control group.^12^ Five domains of bias were assessed for each study including errors in randomization, deviations in group assignments, missing outcome data, measurement of outcome data, and study findings that were reported. The RoB2 tool uses an algorithm that maps response options (yes, probably yes, probably no, and no) to generate a risk-of-bias judgment for each domain. Each study had the option to be rated as low risk, some concerns, or high risk of bias. For non-randomized studies (ie, quasi-experimental without a control group), the Risk of Bias in Non-randomized Studies of Interventions (ROBINS-I) tool was used.^12^^,^^13^ The ROBINS-I tool uses similar algorithms to RoB2 with the primary difference being in the domains assessed. Seven domains of bias for each study were assessed with ROBINS-I including confounding, selection bias, information bias, and reporting bias at pre-, during, and post-intervention. All studies were reviewed for bias by at least one reviewer with a second independent reviewer reviewing 30% of articles to evaluate reliability. Disagreements in ratings were resolved through a discussion with a third reviewer.

### Statistical analysis

Statistical analyses were conducted using IBM SPSS Statistics version 29 (IBM Corp.) and Stata version 19.5 (StataCorp). Due to heterogeneity of raw data reported in the included studies, conversion formulas were used when necessary (ie, converting confidence intervals to SD) to ensure sufficient data were available to calculate effect sizes.^14^ Effect sizes were computed using the Campbell Collaboration web-based effect size calculator.^15^ Several meta-analyses were carried out that were separated by study design (ie, randomized controlled trial (RCT), quasi-experimental with a control group, and quasi-experimental without a control group) as well as by obesity-related outcome, including body mass index (BMI), BMI *z*-score, BMI percentile, waist circumference, and body fat percentage. Quasi-experimental studies were defined as studies that did not implement a “true” randomization scheme. These were further classified into quasi-experimental studies with a control group and quasi-experimental studies without a control group (ie, pretest-posttest design).^16^ Effect sizes (Cohen’s *d*) were calculated as standardized mean differences, with values interpreted as small (0.2-0.5), medium (0.5-0.8), and large (>0.8) effects.^17^ Random-effects models were applied using the inverse-variance weight method to assess intervention effects on BMI, BMI *z*-scores, BMI percentile, waist circumference, and body fat percentage. Heterogeneity among studies was assessed using the *Q*-statistic as well as the restricted maximum likelihood estimation (REML) method and quantified as an *I*-squared (*I*^2^) statistic.^18^ An *I*² value <25% was classified as low heterogeneity, 25% < *I*² < 50% indicated moderate heterogeneity, and ≥50% indicated substantial heterogeneity.^18^

To evaluate the role of potential moderators, random effects subgroup and meta-regression analyses were conducted to assess whether study characteristics were predictors of the intervention effects on obesity outcomes. Differences between categorical subgroup moderators were evaluated using between-group heterogeneity statistics (*Q*-between). Subgroup analyses examined differences by parental involvement (ie, yes or no), country (ie, conducted Mexico vs. US state), border region (ie, whether studies were conducted within 62 miles north and south of the US–Mexico border or not), and intervention target level (ie, individual vs. multilevel). Subgroup analyses were not conducted for obesity outcomes if any category contained fewer than 5 studies. Intervention duration (months) was the only continuous moderator assessed and was examined using random-effects meta-regression models estimated with REML. Funnel plots and Egger’s test were performed for each obesity outcome to assess potential funnel plot asymmetry due to publication bias.

## Results

### Overview of search results

Figure 1 provides a detailed flowchart of the articles screened and retained for final analysis. The initial phase 1 search identified 7,759 records from databases and 912 records from the USand Mexico grey literature. In phase 2, 554 records from databases and 113 records from the grey literature were retained for full-text review. Of these, 476 database records and 101 grey literature records were excluded for not meeting the inclusion and exclusion criteria. In total, 90 studies were included in the systematic review and meta-analysis^19–111^ (Figure 1).

A summary of study characteristics can be located in Material S2. Briefly, most studies were conducted in US border states (79%) including Texas (*n = *30), California (*n = *30), Arizona (*n = *8), and New Mexico (*n = *3) (Material S2). Of the studies that were conducted in Mexican border states (21%), most were conducted in Sonora (*n = *13) and few in Baja California (*n = *2), Nuevo León (*n = *2), or Tamaulipas (*n = *2). Study designs varied and included RCTs (*n = *36, 40%), quasi-experimental studies with a control group (*n = *22, 24%), and quasi-experimental studies without a control group (*n = *32, 36%). On average, studies had a sample size of 282 ± 427.18 participants, with a minimum of 17 participants in one study identified in the grey literature^82^ compared to 3,135 participants in a multi-school RCT.^32^ Overall, studies reported an even distribution of girl to boy participants, with a slight overrepresentation of girl participants (53%).

Interventions were on average a duration of 8.9 months (min: 1 week; max: 5 years) (Material S2). Many of the included studies reported using a theoretical framework (*n = *48, 53%) to guide development of their intervention, targeted multiple levels of the Socioecological Model (*n = *39, 43%), and implemented a combination of physical activity and nutrition behavioral strategies and activities (*n = *86, 96%; see Material S2 for descriptions of intervention activities). Most studies involved parents in the intervention (*n = *71, 79%) and were conducted in multiple settings including schools (*n = *48, 53%), clinics (*n = *19, 21%), community centers (*n = *22, 24%), and participants’ place of residence (*n = *16, 16%). The most common measure of obesity was BMI (*n = *76, 84%) followed by BMIz (*n = *49, 54%), BMI percentile (*n = *48, 53%), waist circumference (*n = *29, 32%), and body fat percentage (*n = *29, 32%). Most studies (*n = *70) recorded height and weight to calculate BMI and BMIz using high-precision medical-grade scales including Tanita (*n = *29), Seca (*n = *30), and Holtain (*n = *6). Several studies (*n = *11) did not report how BMI or BMIz was measured and one study used self-reported height and weight for BMI calculations^111^ (Material S2). Almost all studies (*n = *33) that reported BMI percentile used the Centers for Disease Control (CDC) Growth charts to standardize BMI measurements relative to age- and sex-specific norms. Waist circumference was measured using a flexible tape placed around the abdomen in all studies that reported it as an outcome. However, measurement protocols varied slightly across studies, including differences in rounding conventions (eg, rounding up to the nearest 0.1 or 0.5 cm) and whether values were derived from the average of 2 or 3 measurements. Finally, of the studies that reported body fat percentage, most reported using bioelectrical impedance analysis (*n = *17) followed by dual-energy X-ray absorptiometry (*n = *7), and skinfold thickness (*n = *3).

### Risk of bias

Fifty-eight RCTs and quasi-experimental studies with a control group were assessed for bias; of these, 29 were considered low risk of bias, 18 had some concerns, and 11 had high risk of bias (Figure S1). Most issues were related to the randomization process (*n = *19) and resulted from convenience sampling, unequal distribution of demographic characteristics in the intervention vs. the control group, or self-enrollment of participants. Most studies classified as concerning or high risk of bias were also missing obesity outcome data and excluded from meta-analyses (*n = *11). Of the 32 quasi-experimental studies without a control group assessed for bias; 2 were considered low risk of bias, 13 were moderate, 15 were serious, and 2 were critical risk of bias (Figure S2). The majority of bias was observed due to a lack of measuring and controlling for confounding variables (*n = *23) and missing data or exclusion of participants from analysis without justification (*n = *29).

### Meta-analysis and pooled estimates

#### BMI

Forty-six studies included in the meta-analyses had BMI outcome data; 15 were from Mexican border states while 31 were from US border states. Across all study designs, only one individual quasi-experimental study without a control group reported significant decreases in BMI^89^ (Figure S3). Meta-analytic pooled results for randomized controlled trials (RCTs) (*k* = 17) indicated a small and marginally significant increase in BMI (*d = *0.08, SE = 0.04; 95% CI, −0.00 to 0.17). There were no significant pooled intervention effects on BMI among quasi-experimental studies with a control group (*k = *12) or those without a control group (*k = *17) (Figure S3; Table 1). Substantial between-study heterogeneity was observed across quasi- experimental studies with (*I*^2^ = 96%) and without (*I*^2^ = 81%) control groups, compared to low heterogeneity in RCTs (*I*^2^ = 0%). Across all study designs, there were no statistically significant moderating intervention effects on BMI status by parent involvement, country, border region, intervention target level, or intervention duration (Material S3).

**Table 1:** Meta-analysis results by study design and obesity measure.

	Studies	Cohen’s *d*	SE	95% CI	*Q*_tota1_	*I* ^2^	*P* value	
Randomized controlled trials								
**BMI**	17	0.08	0.04	−0.00 to 0.17	14.05	0	.06	
**BMI-*z***	24	0.01	0.01	−0.01 to 0.03	18.30	0	.48	
**BMI percentile**	10	0.06	0.07	−0.09 to 0.20	26.79	83	.43	
**WC**	12	−0.07	0.05	−0.16 to 0.03	15.67	46	.16	
**% Fat**	10	−0.12	0.07	−0.26 to 0.02	29.01	83	.09	
	*n*	Cohen’s *d*	SE	95% CI	*Q*_tota1_	*I* ^2^	*P* value	
**Quasi-experimental w/control group**								
**BMI**	12	0.28	0.20	−0.11 to 0.67	222.61	96	.16	
**BMI-*z***	11	0.30	0.18	−0.05 to 0.65	146.86	93	.09	
**BMI percentile**	4	0.13	0.07	−0.06 to 0.26	1.28	0	.06	
**WC**	4	0.13	0.11	−0.08 to 0.33	1.66	0	.23	
**% Fat**	4	0.13	0.07	−0.01 to 0.28	1.53	0	.07	
	*n*	Cohen’s *d*	SE	95% CI	*Q*_tota1_	*I* ^2^	*P* value	
**Quasi-experimental w/out control group**								
**BMI**	17	−0.23	0.18	−0.59 to 0.13	97.46	81	.22	
**BMI-*z***	7	0.09	0.09	−0.09 to 0.26	2.06	0	.32	
**BMI percentile**	9	−0.10	0.19	−0.47 to 0.28	0.22	0	.61	
**WC**	9	−0.01	0.12	−0.26 to 0.24	0.49	0	.95	
**% Fat**	6	−0.10	0.22	−0.52 to 0.32	0.17	0	.65	

Abbreviations: BMI = body mass index; CI = confidence interval; *I*^2^ = test of heterogeneity (%); *Q*_total_ = *Q* Statistic; SE = standard error; WC = waist circumference.

##### BMI z-score

In total, 42 studies were included in the meta-analyses for BMI *z*-score, of which 9 studies were from Mexican border states. The overall effect of the interventions on BMI *z*-score was non-significant irrespective of study design with effect sizes ranging from *d* = −0.01 to *d = *0.30 (Figure S4). Substantial heterogeneity was observed across quasi-experimental with a control group study designs (*I*^2^ = 93%) but was low for quasi-experimental without a control group studies (*I*^2^ = 0%) and RCTs (*I*^2^ = 0%; Table 1).

In quasi-experimental studies with a control group, there were statistically significant differences in BMI *z*-score effect sizes by parental involvement (*P* = .03). Interventions that included parental involvement demonstrated no significant changes in BMI *z*-scores (*d* = −0.01; SE = 0.05; 95% CI, −0.12 to 1.26) whereas interventions without parental involvement demonstrated moderate increases in BMI z-scores (*d = *0.67; SE = 0.32; 95% CI, 0.03 to 1.31) (Material S3). No additional moderating effects on BMI *z*-scores were observed by intervention target level or intervention duration (Material S3).

##### BMI percentile

Twenty-three studies reported extractable BMI percentile results. Meta-analytic pooled results for quasi-experimental studies with a control group (*k = *4) indicated a small and marginally significant increase in BMI percentile (*d = *0.13; SE = 0.07; 95% CI, −0.06 to 0.26). RCTs and quasi experimental studies without control group studies demonstrated no significant pooled intervention effects on BMI percentiles (Table 1). Significant heterogeneity was observed across RCT and quasi-experimental without control group study designs (Table 1). There were no significant moderating intervention effects on BMI percentile by intervention duration (Material S3).

#### Waist circumference

Twenty-five studies were included in the meta-analyses for waist circumference. The overall effect of the interventions on waist circumference was non-significant irrespective of study design with effect sizes ranging from *d* = −0.07 to *d = *0.13 (Table 1). Significant heterogeneity was observed across RCTs (Table 1) and there were no significant intervention moderators (Material S3).

#### Percent body fat

Twenty studies were included in the meta-analyses for percent body fat. There were no significant intervention effects on percent body fat across all study designs, with effect sizes ranging between *d* = −0.12 and *d = *0.13 (Table 1). Significant heterogeneity was observed within RCT study designs (Table 1), and there were no significant intervention moderators (Material S3).

### Publication bias results

Results from visual inspection of the funnel plots for each obesity outcome indicated no evidence of publication bias (Figure S5). No statistically significant results from Egger’s regression tests were observed suggesting that funnel plots were symmetrical and that there was no bias due to smaller studies with inflated effect sizes.

## Discussion

This study is the first to our knowledge to systematically evaluate behavioral and multi-level interventions on childhood obesity outcomes among Latino children living in states that border the US and Mexico. Results demonstrated an overall pattern that interventions were not efficacious at reducing childhood obesity, regardless of the type of obesity measure or study design. These results are consistent with a previous global childhood obesity prevention systematic meta-analyses,^112^ which also found no significant effect on obesity outcomes in home, after-school, or community-based interventions. While we observed marginally significant pooled effects on BMI in RCTs as well as on BMI percentile in quasi-experimental studies with a control group, effects were positive and small, indicating that children’s obesity status worsened after completing the intervention. These results are contrary to the proposed hypotheses, but they present an opportunity to consider alternative outcomes that are more amiable and sensitive to change, such as obesity-related behaviors (eg, reducing sedentary behavior, increasing overall PA, and reducing sugary-beverage consumption). Previous studies have demonstrated that obesity-related biomarkers (eg, lipid and glucose profiles, triglycerides, and cholesterol) and physical fitness measures (eg, aerobic fitness, muscular strength, and peak oxygen uptake) among children can be improved through behavioral interventions, and that these changes may be independent of changes in obesity status.^113^^,^^114^ Furthermore, the contradictory findings may also represent an unintended, yet real consequence, of focusing on obesity as the primary study endpoint in children, which may unintentionally result in further exacerbating obesity. For example, previous research has shown that focusing on obesity as the primary outcome in children may contribute to negative body image, psychological distress, weight stigma, and eating disorders.^115^ Thus, future studies targeting childhood obesity in Latino youth in the border region should prioritize improvements in health behaviors and physiological outcomes as measures of intervention effectiveness, rather than obesity itself.

This study also evaluated the potential moderating effects of several factors such as parental involvement, country, border region, intervention target level, and intervention duration. Studies that incorporated parental involvement to reduce obesity were not significant. Interestingly, interventions without parental involvement were associated with moderate increases in BMI *z*-scores, suggesting that while parental involvement may not lower BMI, it could help prevent increases in BMI. These results are partially supported by previous studies which demonstrate that parental involvement positively impacts child PA, dietary behaviors, and obesity outcomes.^116^ However, given that almost all other potential moderators tested were non-significant and numerous statistical tests were conducted, there is a probability this result was due to a type 1 error, rather than a true positive result.

There are several notable patterns discovered in this study. First, most studies (60%) were quasi-experimental study designs. This may partially explain the overall non-significant results given the methodological issues related to these non-randomized study designs. However, even among RCT designs, the pooled results were mostly non-significant, suggesting that study design does not change the pattern of effects. Second, the results did not differ based on the type of obesity measure (eg, BMI, BMIz, waist circumference). In fact, the majority of effect sizes across all obesity measures can be classified as “small.” These findings are in contrast to a recent meta-analysis among Hispanic youth showing that childhood obesity focused interventions significantly decreased BMI and waist circumference.^8^ However, this meta-analysis included studies conducted throughout the US whereas the current study focused on interventions implemented in US and Mexico border states. Differences in findings may reflect unique contextual factors such as limited regional resource availability, cultural practices, and cross-border influences that may be impacting intervention delivery and efficacy in interventions conducted near the US–Mexico border. Further studies in the border region are needed to determine whether binational strategies are needed to improve obesity outcomes within border region populations.

Finally, of the 90 total studies included in this systematic review, only 19 studies were conducted in Mexico, and no studies were conducted concurrently in the US and Mexico. This represents an opportunity and a call to action to conduct more binational research in this area, particularly among communities living in the US–Mexico border region. In addition, it may be important to acknowledge that obesity prevention could benefit from a broader conceptualization beyond individual and family behavioral change, as some researchers have proposed.^117^^,^^118^ Few studies were identified that conducted interventions addressing structural or policy levels of change. However, the food, social, and built environment are crucial mediators of behavior that can either facilitate or hinder an individual’s motivation for change and agency, especially in a binational context.^119^ The lack of significant improvements in obesity outcomes observed in the current study indicates an urgent need for the improvement of environments for healthy eating and physical activity, implementation of fiscal policies, regulation of food marketing and labeling, and monitoring and evaluation of these actions in the US–Mexico border region to address childhood obesity.^118^

### Strengths and limitations

Strengths of the current study include a comprehensive review of US, Mexico, and global databases to identify eligible studies, the inclusion of grey literature, and evidence of no publication bias. Procedures and bias assessment were conducted using validated instruments and multiple reviewers to ensure consistency. Limitations include a focus on measures of obesity rather than behavioral outcomes (eg, improvements in physical activity or dietary intake), high heterogeneity observed in the studies, and multiple meta-analyses and moderator tests which may have increased the possibility of type 1 errors. Another limitation was the low number of interventions conducted in the border region or for longer than 12 months. This was mitigated by expanding the search to border states and conducting a moderator analysis by border region classification and intervention duration. There were several other moderators that were not assessed including setting (ie, school-based vs. community-based interventions), socioeconomic status of participants, developmental phase (children vs. adolescents), and measurement type. These were not included to minimize the risk of a type 1 error. Lastly, no studies were identified in the border Mexican states of Chihuahua or Coahuila. Consequently, we were unable to assess the efficacy of interventions in these regions, and findings in the current review may not be generalizable to these states.

## Conclusion

The present review is the first known study to assess the efficacy of childhood obesity interventions at the US–Mexico border. Results demonstrated null to minimal changes in obesity outcomes regardless of study design or location. Meta-regression results suggest that parental involvement may prevent increases in BMI. Few studies were conducted in Mexico and no studies implemented binational strategies. Considering that the US–Mexico border is a unique environment for obesity prevention efforts due to a fluid exchange of ideas, language, cultural practices, social norms, and economic activities, a coordinated binational and tailored obesity prevention effort may be more appropriate for populations residing in this region.

## Supplementary Material

kaag056_Supplementary_Data

## Data Availability

Data from this study are not available in a public archive. Data from this study will be made available by emailing the corresponding author. Analytic code used to conduct the analyses presented in this study are not available in a public archive. They may be available by emailing the corresponding author.

## Study registration

This study was registered after the study began. The study is registered at International Platform of Registered Systematic Review and Meta-analysis Protocols (INPLASY202440046).

## Analytic plan pre-registration

The analysis plan was registered after data collection commenced and is registered at (INPLASY202440046).

## Materials availability

Materials used to conduct the study are not publicly available.
