# Supplementary material for: Childhood obesity interventions among Latinos residing in the US–Mexico border: a systematic review and meta-analysis of obesity outcomes
Source: Ann Behav Med. 2026 Sep 16;60(1):kaag056. doi: 10.1093/abm/kaag056 (PMC13581397; doi:10.1093/abm/kaag056)
Supplement: Supplementary file 1 [file kaag056_supplementary_data.zip › XXXXXSupplementary_data.zip!/Supplementary Material 1.docx]

**Supplementary Material 1**

**Database Search Keywords**

*Child Obesity:* obesity; At risk for overweight; BMI; skin fold thickness; adiposity outcomes; rate of weight gain; weight gain; weight loss; body weight; percent fat content; weight and height; waist circumference; waist-to-hip ratio, abdominal obesity, percent body fat; overweight; obese; overfat; high fat; subcutaneous fat; visceral fat; intra-abdominal fat; abdominal fat; trunk fat; IAAT (intra-abdominal adipose tissue); heavy; BMI percentile; BMI Z-score; BMI category; weight trajectory; obesity rebound; excess fat; morbidly obese; massively obese; body fat distribution; fat mass; adipose tissue; quetelet index.

*Pre-school and school-age:* children or child or preschoolers or pre-adolescent or school-age or childhood.

*Race/ethnicity (search in the United States):* Latino or Latinas or Latinx or Hispanics or Chicanos or Chicanas or Mexican or Mexican American or Spanish-speaking or Latin immigrant.

*Physical Activity:* Physical activity, exercise, aerobic exercise, exercise training, fitness, capacity fitness, physical fitness, movement, cardiovascular fitness, sports, resistance training, endurance training, acute exercises, physical exertion, motor activity, physical functioning, isometric exercise, physical conditioning, gardening, steps, walking, leisure-time activity, moderate-to-vigorous physical activity

*Sedentary Behavior:* sedentary behavior, sedentary lifestyle, physical inactivity, lack of physical activity, sedentary time, screen time, watching TV, computer, playing video games, sitting

*Diet and nutrition:* Nutrition, diet, nutritional status, healthy diet, healthy eating, dietary behavior, eating, dietary intake, nutritional intake, portion size, serving size, food and beverage, sugar-sweetened beverages, fruit, vegetables, grains, family meals, feeding behavior, food habits, eating habits, fast food, cooking, junk food, food insecurity, food security, food dessert, food swamps, food environment, food access, food availability, dietary practices, food purchasing, food beliefs, healthy eating index

*Study Design:* Controlled clinical trials; clinical trial; randomized controlled trial; randomized clinical trial; randomized trial; group randomized trial; randomization; double-blind procedure; single-blind procedure; crossover procedure; comparative study; matched communities/matched schools/matched populations; comparison group; control group; matched pairs; outcome study/studies; quasi-experimental/pseudo experimental; nonrandomized/pseudo-randomized/quasi-randomized; treatment & placebo; treatment; intervention; experimental; pre-post design; before-after; prospective cohort study; retrospective cohort study; case-control; time series; adaptive trial, pragmatic trial, MOST design, step-wedge design, single-case study design, time-series design, interrupted time-series design, factorial design, longitudinal.

**Grey literature Keywords**

**Keywords**

- The search within ProQuest included ‘Latino children and obesity and (intervention or randomized control trial) and (California or Texas or New Mexico or Arizona’. Additional ProQuest searches were conducted with adiposity-related terms such as ‘BMI’, ‘at risk for overweight’, and ‘skin fold thickness.’
- The search in SSRN included ‘childhood obesity' and ‘program’, ‘childhood obesity’ and ‘intervention’, as well as ‘obesity’ and ‘program’ with ‘child’.
- For UNAM, ‘childhood obesity’ with each of the following locations were separately searched: Baja California, Sonora, Chihuahua, Coahuila, Tamaulipas, and Nuevo Leon.
- The APHA conference search included abstracts and programs with the terms ‘childhood obesity’, ‘intervention or program’, and ‘California’ (or ‘Texas’ or ‘Arizona’ or 'New Mexico’).
- In Worldcat, searches were conducted with the terms ‘Latino children’ and ‘obesity’ and ‘program or intervention’, as well as searches including California, Arizona, Texas, and New Mexico.
- The PCD collection searches included ‘childhood obesity’ and ‘intervention or program’ and ‘California or Texas or Arizona or New Mexico’.
- The search within the Repositorio Institucional Unison included "childhood obesity" and "program or intervención" while "childhood obesity" and "program" were searched in CIAD.
- In the Universidad de Navojoa, Universidad Autónoma de Nuevo León, Universidad de Autónoma de Ciudad Juárez, Universidad de Montemorelos, the term ‘childhood obesity’ was searched in combination with the terms ‘program’, ‘intervention’, ‘nutrition, ‘physical activity’, ‘Baja California’, ‘Sonora’, “Chihuahua’, ‘Coahuila’, ‘Nuevo León’, and Tamaulipas’.
- In COLEF, ‘childhood obesity’ and ‘program or intervention’ were searched.
- For searches within AMMFEN, Congresos del Colegio Mexicano de Nutriólogos, INSP, the following terms were utilized: ‘obesidad, infantil, obesidad infantil, preescolar, escolar, actividad física, sedentario, sedentarismo, obesidad escolar, nutricion, nutricion escolar.’
