# Supplementary material for: Childhood obesity interventions among Latinos residing in the US–Mexico border: a systematic review and meta-analysis of obesity outcomes
Source: Ann Behav Med. 2026 Sep 16;60(1):kaag056. doi: 10.1093/abm/kaag056 (PMC13581397; doi:10.1093/abm/kaag056)
Supplement: Supplementary file 1 [file kaag056_supplementary_data.zip › XXXXXSupplementary_data.zip!/Supplemental Material 2_FINAL.docx]

**Supplemental Material 1.** Summary of Records Included in Systematic Review and Meta-Analysis

| **First Author, Year** | **Location** | **Study Design** | **Sample Size** | **Age (years)** | **Percent Female (%)** | **Intervention Description** | | | | **Obesity Outcomes Reported and Measures Used** |
| --- | --- | --- | --- | --- | --- | --- | --- | --- | --- | --- |
|  |  |  |  |  |  | **Intervention Strategies** | **Duration / Frequency** | **Parent Involve-ment**  **(Y/N)** | **Socioecological Model Level & Setting** | **BMI:**  **BMIz:**  **BMI percentile:**  **WC:**  **Body Fat%:** |
| Aguirre et al., 2025 [19] | Los Angeles, California, USA | Quasi w/ Control | Int N = 91  Ctrl N = 85 | 8-9 | ^44.8^ | **Program Name:** *Familias Saludables*  **Theory:** Health Belief Model (HBM) and Social Cognitive Theory (SCT)  **Channels delivered:** Trained bilingual graduate research fellows in Public Health and Kinesiology master’s programs as well as bilingual community health workers.  **Activities:** Sessions included family-based bilingual PA and nutrition activities. Each session included a 60-min interactive family health education module followed by 30 min of PA for the children (based on the SPARK curriculum) while parents received health coaching. Sessions concluded with 20 min of family goal setting. | 6 weeks  Weekly 120-minute sessions | Y | **Level:** Family, Community  **Setting:** Community Center | **BMI:** Tanita digital scale  **BMIz:** N  **BMI percentile:** Center for Disease Control (CDC) Growth Charts  **WC:** N  **Body Fat %:** Biolectrical impedance (Tanita) |
| Alexander, Grant, Pedrino, and Lyons, 2014 [20] | Palm Springs, California, USA | RCT | Int N = 272  Ctrl N = 289 | 6-8 | ^a^NR | **Program Name:** ^a^NR  **Theory:** ^a^NR  **Channels delivered:** Physical education specialists, schoolteachers  **Activities**: Structured daily recess time with extra exercises, one extra physical education class, after-school cooking classes, assemblies with nutritional and recreational topics, nutritional education, seminars for teachers and administrators, “Chef in the Classroom” program, “Health Camps” intersessions, parent counseling sessions, and monthly parent meetings. | 12 months | Y | **Level:** Community  **Setting:** School | **BMI:** Stadiometer and weight-measuring scale  **BMIz:** N  **BMI percentile:** National Center for Health Statistics (NCHS) percentiles  **WC:** N  **Body Fat %:** N |
| Alexander & Lyons, 2016 [21] | Palm Springs, California, USA | Quasi w/out Control | N = 183 | 6-8 | ^a^NR | **Program Name:** ^a^NR  **Theory**: ^a^NR  **Channels delivered:** Physical education specialists, nutritionists  **Activities:** Physical education, cooking classes, nutritional classes, and parental counseling. | 24 months | Y | **Level:** Family  **Setting:** School, home | **BMI:** Tanita digital scale and weight-measuring scale  **BMIz:** N  **BMI percentile:** QuesGen Systems online calculator  **WC:** N  **Body Fat %:** N |
| Álvarez Fariña et al., 2020 [22] | Hermosillo, Sonora, MX | Quasi w/out Control | N = 321 | 8-11 | 53 | **Program name:** *Por tu Salud*  **Theory:** ^a^NR  **Channels delivered:** Physical education teacher  **Activities:** Physical activity, school-based program. | 3 months; 3 days/week of 60-min PA | N | **Level:** Individual  **Setting:** School | **BMI:** Stadiometer (Microtoise) and weight-measuring scale (Seca)  **BMIz:** N  **BMI percentile:** N  **WC:**  Flexible Tape  **Body Fat %:** N |
| Bacardi-Gascon, Pérez-Morales, & Jiménez-Cruz, 2012 [23] | Tijuana, Baja California, MX | RCT | Int N = 271  Ctrl N = 244 | 8.5±0.7 | 48 | **Program Name:** ^a^NR  **Theory**: Bronfenbrenner’s Ecological Model  **Channels delivered:** Nutrition and physical activity professionals  **Activities:** School board and teacher involvement, healthy lifestyle classes. | 8 months; Kids: weekly 30-minute sessions, Parents: monthly 60-min session | Y | **Level:** Community  **Setting:** School | **BMI:** Stadiometer (Seca) and weight-measuring scale (Tanita)  **BMIz:** WHO LMS Parameters  **BMI percentile:** N  **WC:** Flexible Tape (SECA)  **Body Fat %:** N |
| Bender et al., 2013 [24] | San Diego, California, USA | Quasi w/out Control | N = 33 mother-child dyads | 3.6±0.7 | 52 | **Program Name:** *Vida Saludable* (Healthy Living)  **Theory:** Social Cognitive Learning Theory  **Channels delivered:** *Promotora*  **Activities:** Interactive group lessons; group community activities (e.g. local field trips to grocery stores); physical activity sessions. | 9 months; biweekly sessions for 2 months followed by 6 monthly group sessions | Y | **Level**: Individual, Family  **Setting**: Clinic, Community | **BMI:**  Height and weight-measuring scale  **BMIz:** N  **BMI percentile:** CDC Growth Charts  **WC:** N  **Body Fat %:** N |
| Briones-Villalba et al., 2018 [25] | Tijuana, Baja California, MX | Quasi w/ Control | Int N = 21  Ctrl N = 14 | Int: 9.5±1.1 Control: 9.0±1.5 | 65 | **Program Name:** *Cimarrones*  **Theory:** ^a^NR  **Channels delivered:** ^a^NR  **Activities:** Warm-up for physical activity sessions, circuit exercises, 15-minute cool-down sessions during which mini nutrition topics were discussed; parents completed 2 nutrition-related workshops. | 9 months; 3 weekly 60-min sessions | Y | **Level**: Individual, Family  **Setting**: School | **BMI:** Stadiometer (Seca) and weight-measuring scale (Tanita)  **BMIz:** N  **BMI percentile:** N  **WC:** Flexible Tape (SECA)  **Body Fat %:** N |
| Butte et al., 2017 [26] | Houston, Texas, USA | RCT | Int N = 315  Ctrl N = 234 | 3 age groups: 2-5, 6-8, and 9-12 | 52 | **Program Name:** Texas Childhood Obesity Demonstration (TX CORD)  **Theory**: Principles of nutritional and sports science, learning, and Social Cognitive Theory  **Channels delivered:** Bilingual Allied Health Professionals  **Activities**: Physical activity sessions, CATCH activities, cooking classes, the “Being Well” book of healthy strategies for families, and the MEND World website. | 12 months; 9 weekly 90-min sessions for 3 months followed by monthly reinforcement sessions for 9 months | N | **Level:** Individual  S**etting:** Clinic | **BMI:**  Height and weight-measuring scale  **BMIz:** N  **BMI percentile:** N  **WC:** N  **Body Fat %:**  Biolectrical impedance (Tanita) |
| Coleman et al., 2010 [27] | San Diego, California, USA | Quasi w/out Control | N = 62 | 7.5±3.1 | 53 | **Program Name:** Horton Hawks Stay Healthy  **Theory:** ^a^NR  **Channels delivered:** ^a^NR  **Activities:** Physical activity sessions, cooking demonstrations, parent-child lectures on barriers to healthy lifestyle decisions and portion sizes. | 2.5 months; weekly 90-min sessions | Y | **Level**: Family  **Setting**: School | **BMI/BMIz:** Height and weight-measuring scale  **BMI percentile:** ^a^NR  **WC:** N  **Body Fat %:** N |
| Coleman et al., 2005 [28] | El Paso, Texas, USA | Quasi w/ Control | Int N = 423 Ctrl N = 473 | 8.3±0.5 | 47.9 | **Program Name:** Child and Adolescent Trial for Cardiovascular Health [CATCH]  **Theory:** ^a^NR  **Channels delivered:** PE teachers and food service staff  **Activities:** PE classes, healthy meals provided to cafeterias, and classroom curriculum. | 4 years | N | **Level:** Individual, Community  **Setting:** School | **BMI:** Height and weight-measuring scale  **BMIz:** N  **BMI percentile:** N  **WC:** N  **Body Fat %:** N |
| Costa-Urrutia et al., 2019 [29] | Hermosillo, Sonora, MX | Quasi w/out Control | N = 320 | 8.2±2.3 | 49 | **Program Name:** ^a^NR  **Theory:** ^a^NR  **Channels delivered:** Physical education teachers, psychologists, and nutritionists  **Activities:** Physical activity and health education sessions, parent workshops, mid-morning playtime, and lunch at school. | 3 months; 3 60-min PA sessions per week; one 50-min weekly health education session | Y | **Level**: Individual  **Setting**: School | **BMI/BMIz:** Stadiometer (Microtoise) and weight-measuring scale (Seca)  **BMI percentile:** N  **WC:** N  **Body Fat %:** N |
| Crespo et al., 2012 [30] | San Diego, California, USA | RCT | Int N = 481 Ctrl N = 227 | 5.9±0.9 | 50 | **Program Name:** *Aventuras Para Niños*  **Theory:** Health Belief Model, Social Cognitive Theory, Structural Model of Health Behavior  **Channels delivered:** *Promotoras*  **Activities:** Physical education, changes to physical education equipment, and healthy children’s menus in restaurants, posters, and newsletters on healthy eating. | 3 years; 7 annual home visits, 4 booster calls | Y | **Level:** Individual, Family, Community  **Setting:** Family, community center, local restaurants | **BMI:** Height and weight-measuring scale  **BMIz:** CDC Growth Charts  **BMI percentile:** CDC Growth Charts  **WC:** N  **Body Fat %:** N |
| Crespo et al., 2018 [31] | San Diego, California, USA | RCT | Int N = 481 Ctrl N = 227 | 7.6±1.5 | 50 | **Program name:** *Luces de Cambio*  **Theory:** Socioecological Model for Latino Health Promotion  **Channels delivered:** Bilingual Health Educators  **Activities:** A traffic light concept was taught, where healthy activity and food groups were green, group classes, visits to the clinic with a provider, scripted phone calls, and group booster classes. | 12 months;  Seven 1.5 – 2-hour group classes | Y | **Level**: Individual, Family  **Setting**: Home | **BMI:** Height and weight-measuring scale  **BMIz:** CDC Growth Charts  **BMI percentile:** CDC Growth Charts  **WC:** N  **Body Fat %:** Dual-energy X-ray Absorptiometry (DXA) |
| Crespo et al., 2025 [32] | Phoenix, Arizona, USA | RCT | Int N = 69 Ctrl N = 68 | 9.3±1.7 | 58.4 | **Program Name:** Athletes for Life (AFL)  **Theory:** Social Cognitive Theory, Operant Learning Theory, Socioecological Model  **Channels delivered:** Bilingual Latino facilitators  **Activities:** Community-and family-based behavioral intervention designed to increase cardiovascular fitness and improve dietary quality through progressive intensity programming focused on sports skills and health behavior modification among both parents and their children. | 12 weeks; Two weekly 90-minute sessions | Y | **Level:** Family, Community  **Setting:** Community Center | **BMI:** Height and weight-measuring scale  **BMIz:** CDC Growth Charts  **BMI percentile:** CDC Growth Charts  **WC:** ^a^NR  **Body Fat %:** Dual-energy X-ray Absorptiometry (DXA) |
| Cruz et al., 2016 [33]^b^ | Hermosillo, Sonora, MX | Quasi w/out Control | N = 20 | 8-11 | ^a^NR | **Program Name:** ^a^NR  **Theory:** ^a^NR  **Channels delivered:** Students  **Activities:** Exercises (e.g., curl-ups, cardio, etc). | 5 months; bi-weekly sessions | N | **Level**: Individual  **Setting**: School | **BMI:** Height and weight-measuring scale (Seca)  **BMIz:** N  **BMI percentile:** N  **WC:** Flexible Tape  **Body Fat %:** Dual-energy X-ray Absorptiometry (DXA; Hologic Discovery Wi) |
| Davis et al., 2011 [34] | Los Angeles, California, USA | Quasi w/ Control | Int N = 34  Ctrl N = 70 | 9.8±0.7 | 48 | **Program name:** LA Sprouts (pilot study)  **Theory:** ^a^NR  **Channels delivered**: Dietitian  **Activities:** Sessions combined gardening, nutrition education, cooking at a community garden, and optional parent-focused nutrition classes. | 3 months; weekly 90-min sessions | Y | **Level:** Individual  **Setting:** School | **BMI/BMIz:** Height (Perspective) and weight-measuring scale (Tanita)  **BMI percentile:** CDC Growth Charts  **WC:** Flexible Tape  **Body Fat %:** Biolectrical impedance (Tanita) |
| Davis et al., 2016 [35] | Albuquerque, New Mexico, USA | RCT | Int N = 500  Ctrl N = 480 | 2-5 | 47.4 | **Program Name:** Child Health Initiative for Lifelong Eating and Exercise (CHILE)  **Theory:** Socioecological Model  **Channels delivered:** School teachers and local healthcare providers  **Activities**: Nutritional health education and physical activity sessions incorporated into curriculums, teacher & food service training, healthy food policies, family engagement, and grocery store partnerships. | 24 months; Daily 30-min sessions | Y | **Level**: Family, Community  **Setting**: Community center, Schools | **BMI:** Height (Shorr board) and weight-measuring scale (Seca)  **BMIz:** CDC Growth Charts  **BMI percentile:** CDC Growth Charts  **WC:** N  **Body Fat %:** N |
| Davis et al., 2021 [36] | Austin, Texas, USA | RCT | Int N = 8 schools, 1412 students  Ctrl N = 8 schools, 1723 students | 9.2±0.1 | 50 | **Program Name:** Texas Sprouts  **Theory:** ^a^NR  **Channels delivered:** Garden/nutrition educators  **Activities:** School-based gardening, nutrition health education, cooking program, and parent sessions. | 12 months; 18 one-hour lessons throughout the year | Y | **Level:** Individual  **Setting:** School | **BMI:** Height (Seca) and weight-measuring scale (Tanita)  **BMIz:** CDC Growth Charts  **BMI percentile:** CDC Growth Charts  **WC:** Flexible Tape  **Body Fat %:** Biolectrical impedance (Tanita) |
| de Heer et al., 2011 [37] | El Paso, Texas, USA | RCT | Int N = 242  Ctrl N = 354 | 9.2 ±1.0 | 47 | **Program Name:** ^a^NR  **Theory:** Social Cognitive Theory  **Channels delivered:** Senior-level student teachers, Physical Education teachers, and community health workers  **Activities**: Health education on healthy eating, exercise, and self-esteem; physical activity curriculum focused on cardiovascular activity and aerobic recreational games. | 3 months; bi-weekly 90-min sessions | N | **Level:** Community  **Setting:** School | **BMI:** Tanita digital scale  **BMIz:** N  **BMI percentile:** ^a^NR  **WC:** N  **Body Fat %:** N |
| Delcid, 2013 [38]*^b^* | Hermosillo, Sonora, MX | Quasi w/out Control | N = 59 | 6-11 | 54 | **Program Name:** Programa de Atención al Sobrepeso y Obesidad en Sonora (PASOS)  **Theory:** ^a^NR  **Channels delivered**: Students  **Activities**: Physical and nutritional health education sessions. | 6 months | N | **Level:** Individual  **Setting:** School | **BMI:** N  **BMIz:** Height (Holtain) and weight-measuring scale (Seca)  **BMI percentile:** ^a^NR  **WC:** Flexible Tape (Gulick)  **Body Fat %:** Dual-energy X-ray Absorptiometry (DXA; Hologic Discovery Wi) |
| Díaz et al., 2010 [39] | Hermosillo, Sonora, MX | RCT | Int N = 21  Ctrl N = 22 | 11.7±2.2 | 53 | **Program Name:** *Programa Cambia*  **Theory:** ^a^NR  **Channels delivered**: Registered dietitian and primary care physician  **Activities:** Nutrition education sessions with parents and kids, family support, and weekly consultation with a nutritionist. | 3 months; weekly 120-min sessions | Y | **Level**: Individual, Family  **Setting**: Clinic | **BMI:** Height (Holtain) and weight-measuring scale (FV-150)  **BMIz:** Epi Info Software  **BMI percentile:** N  **WC:** ^a^NR  **Body Fat %:** Dual-energy X-ray Absorptiometry (DXA; Lunar DPX-MDi) |
| Dreimane et al., 2007 [40] | Los Angeles, California, USA | Quasi w/out Control | N = 264 | 11.6±2.2 | 52 | **Program Name:** Kids N Fitness  **Theory:** ^a^NR  **Channels delivered:** Registered dietitians and physical therapists  **Activities:** Participated in 45-minute physical activity sessions, followed by a 45-minute nutrition education and behavioral modification session. | 8-12 weeks; weekly 90-min sessions | Y | **Level:** Family  **Setting:** Clinic | **BMI:** Height (Harpenden) and weight-measuring scale (Detecto)  **BMIz:** Epi Info Software  **BMI percentile**: N  **WC:** N  **Body Fat %:** N |
| Early & Cheffer, 2019 [41] | Long Beach, California, USA | Quasi w/ Control | Int N = 15  Ctrl N = 20 | 10.8 | 54.4 | **Program Name:** 5-2-1-0 Let's Go! Program  **Theory:** Social Ecological Model (SEM)  **Channels delivered:** Pediatrician or nurse practitioner  **Activities:** Motivational interviewing to identify 1 health behavior the family wanted to change, home visits to reinforce MI. | 6 months; 3 home visits | Y | **Level:** Individual and Family  **Setting:** Clinic and home | **BMI:** ^a^NR  **BMIz:** N  **BMI percentile**: CDC Growth Charts  **WC:** N  **Body Fat %:** N |
| Falbe et al., 2015 [42] | Contra Costa County, California, USA | RCT | Int N = 26  Ctrl N = 27 | 8.9±1.8 | 51 | **Program Name**: Active and Healthy Families (AHF)  **Theory:** Transtheoretical Model  **Channels delivered:** Dietitian, physician, and *promotoras*  **Activities:** Health education on nutrition, review of medical records, role play between child and parent, and outdoor play. | 2.5 months; biweekly group sessions | Y | **Level:** Family  **Setting**: Home | **BMI:** Height (420 Measure) and weight-measuring scale (Tanita)  **BMIz:** Y  **BMI percentile:** N  **WC:** N  **Body Fat %:** Biolectrical impedance (Tanita) |
| Feng et. al., 2016 [43] | Lubbock, Texas, USA | Quasi w/ Control | Int N = 332  Ctrl N = 265 | 6.7±1.0 | 53 | **Program Name:** ^a^NR  **Theory:** Community-based  Participatory research approach  **Channels delivered:** Trained instructors, class-  room teachers, physical education teachers, and certified  Community health workers  **Activities:** Nutrition education, exercise, gardening, and home visits; take-home workbooks and information sheets for parents. | 18 months; 9 randomly selected classes as intervention | Y | **Level:** Family  **Setting:** School, Home | **BMI/BMIz:** Tanita digital scale  **BMI percentile:** CDC Growth Charts  **WC:** ^a^NR  **Body Fat%:** N |
| Foster et al., 2016 [44] | Cameron County, Texas, USA | RCT | Int N = 23  Ctrl N = 25 | 4.5±0.65 | 11 | **Program Name:** Neighbors in Need of Services (NINOS)  **Theory:** Positive deviance approach  **Channels delivered:** Intervention group: parent mentors trained in positively deviant behaviors regarding childhood obesity. Comparison group: community health workers providing education on obesity-related behaviors.  **Activities:** Home visits to families, phone calls, and community meetings at Head Start centers. | 6 months | Y | **Level**: Individual, Family, and Community  **Setting**: Home and clinic | **BMI:** Height and weight-measuring scale  **BMIz:**  CDC Growth Charts  **BMI percentile: N**  **WC: N**  **Body Fat%: N** |
| Fullerton, et. al., 2007 [45] | Houston, Texas, USA | Quasi w/ Control | ^a^NR | 12.1 | ^a^NR | **Program Name:** ^a^NR  **Theory**: ^a^NR  **Channels delivered:** ^a^NR  **Activities**: Nutrition and physical activity classes and behavior strategies such as self-monitoring, goal setting, and controlling environmental cues. | 6 months; weekly sessions for 3 months followed by monthly sessions. | N | **Level**: Community  **Setting**: School | **BMI:** N  **BMIz:** Tanita and CDC Growth Charts  **BMI percentile:** N  **WC:** N  **Body Fat%:** N |
| Garcia et al., 2022 [46]^b^ | Monterrey, Nuevo León, MX | Quasi w/out Control | N = 41 | 4.29±0.9 | ^a^NR | **Program Name:** *e-Cambio Saludable*  **Theory:** Social Cognitive Theory  **Channels delivered:** *Promotoras*  **Activities:** Sessions aimed to improve maternal perception of their child's weight, feeding goals, and healthy infant feeding practices through an online platform. | 1 month;  weekly 50-min modules | Y | **Level:** Family  **Setting:** ^a^NR | **BMI:** Height (SECA 213) and weight-measuring scale (SECA)  **BMIz:** N  **BMI percentile:** Organización Mundial de la Salud 2010 Classifications  **WC:** N  **Body Fat%:** N |
| Gatto et al., 2017 [47] | Los Angeles, California, USA | RCT | Int N = 172  Ctrl N = 147 | 9.3±0.9 | 52 | **Program Name:** LA Sprouts (RCT)  **Theory:** Bandura’s “self-efficacy” construct  **Channels delivered:** Health educator  **Activities**: “Hands-on” and interactive classes targeting cooking, gardening, and nutrition. | 3 months; weekly 80-min sessions | Y | **Level**: Individual, Family  **Setting**: School | **BMI:** N  **BMIz:** Height (Seca) and weight-measuring scale (Tanita)  **BMI percentile:** CDC Growth Charts  **WC:** Flexible Tape  **Body Fat %:** Biolectrical impedance (Tanita) |
| Gimpel et al., 2010 [48] | Dallas County, Texas, USA | Quasi w/out Control | N = 34 | 10.4±2.1 | ^a^NR | **Program Name:** Fitness in Nutrition and Exercise (FINE)  **Theory:** ^a^NR  **Channels delivered:** Pediatrician, nurse, nutritionist, medical assistant, interpreters, public health educator, and pediatric social worker  **Activities:** SMA consists of four components: (1) medical history and vital signs; (2) nutrition education; (3) social issues; and (4) physical activity. | 4 months; monthly 30-min sessions | Y | **Level:** Family  **Setting**: Clinic | **BMI:** ^a^NR  **BMIz:** N  **BMI percentile:** N  **WC:** N  **Body Fat:** N |
| Gómez-Plata & Ayala, 2014 [49]*^b^* | Hermosillo, Sonora, MX | Quasi w/out Control | N = 35 | 9.9±2.1 | 47.2 | **Program Name:** ^a^NR  **Theory:** ^a^NR  **Channels delivered:** Undergraduate students in sports, nutrition, nursing, and psychology from the University of Sonora  **Activities**: The first half of the session was dedicated to physical activity and the last half hour was a workshop on Nutrition, Health Risk Prevention, Physiotherapy, or Psychology. | 12 months; daily 2-hour sessions | Y | **Level**: Individual  **Setting**: ^a^NR | **BMI:** Height (SECA 213) and weight-measuring scale (Tanita)  **BMIz:** World Health Organization 2010 Classifications  **BMI percentile:** N  **WC:** Flexible Tape (Gulick)  **Body Fat %:** Biolectrical impedance (Tanita) |
| González-Heredia et al., 2014 [50] | Obregon, Sonora, MX | Quasi w/ Control | Int N = 30  Ctrl N = 30 | 9.2±2.1 | 51.6 | **Program Name:** ^a^NR  **Theory:** ^a^NR  **Channels delivered:** ^a^NR  **Activities:** The intervention consisted of health education on eating habits in schoolchildren and their parents. | 6 months | Y | **Level**: Family  **Setting**: Clinic | **BMI:** Height and weight-measuring scale  **BMIz:** N  **BMI percentile:** N  **WC:** N  **Body Fat %:** N |
| González-Ramírez & Pompa-Guajardo, 2011 [51] | Nuevo Leon, MX | Quasi w/ Control | Int N = 35  Ctrl N = 33 | 8.8–10.1 | 23.8**-**42.9 | **Program Name:** *Vacaciones Saludables*  **Theory:** ^a^NR  **Channels delivered:** Specialists in nutrition, psychology, pediatrics, and sports medicine  **Activities:** A multidisciplinary program that began with a summer camp and then, monthly and biweekly sessions that included a controlled exercise program, a balanced diet, and sessions with a psychologist. | 6 months; monthly 90-min sessions | Y | **Level:** Individual, Family  **Setting:** ^a^NR | **BMI:** Height and weight-measuring scale (Tanita)  **BMIz:** N  **BMI percentile:** N  **WC:** N  **Body Fat %:** N |
| Guerrero et al., 2023 [52] | Los Angeles, California, USA | Quasi w/out Control | N = 54 | 3.8 | ^a^NR | **Program Name:** *Familias Unidas, Niños Sanos* (FUNS)  **Theory:** Family Systems Approach  **Channels delivered:** Trained health educators  **Activities:** Traditional in-person group sessions were combined with eight weeks of mobile phone content to target parenting skills and improve age-appropriate nutritional practices. The Chorus platform was a web-based application used to deliver FUNS content. | 6 months; weekly 1- hour session | Y | **Level:** Family  **Setting:** Community Center | **BMI:** NHANES Anthropometry Procedure  **BMIz:** N  **BMI percentile:** CDC Growth Charts  **WC:** N  **Body Fat %:** N |
| Hawthorne et al., 2011 [53] | Phoenix, Arizona, USA | Quasi w/out Control | N= 1074 | 5-11 | 51 | **Program Name:** Grand Canyon Trekkers  **Theory:** ^a^NR  **Channels delivered:** Research team and parent volunteers  **Activities**: Walk during lunchtime recess, incentives, certificates, and program opportunities. | 4 months  3 days per week, 25 minutes session | N | **Level:** Individual  **Setting**: School | **BMI:** Height (SECA) and weight-measuring scale (Tanita)  **BMIz:** N  **BMI percentile:** CDC Growth Charts  **WC:** Flexible Tape  **Body Fat %:** Biolectrical impedance (Tanita) |
| Hingle, 2008 [54]*^b^* | Tucson, Arizona, USA | Quasi w/ Control | Int N = 33  Ctrl N = 16 | 9.2±0.6 | 53.5 | **Program Name:** *Activa Y Sana*  **Theory:** Social Learning Theory; Theory of Planned Behavior; Social Ecological Model  **Channels delivered:** School teachers, nutrition educators, YMCA staff, and university staff  **Activities:** Three intervention conditions. Level 1: state-mandated  nutrition- and physical activity-based health curriculum; Level 2: Level 1 curricula plus an after-school program; Level 3: Level 1 + Level 2 activities, plus a family program. | 48 months | Y | **Level:** Individual, Family  **Setting**: School, Community | **BMI/BMIz:** Height (Shorr) and weight-measuring scale (Seca)  **BMI percentile:** N  **WC:** Flexible Tape  **Body Fat %:** Biolectrical impedance (RJL Systems) |
| Hughes et al., 2020 and Hughes et al., 2021 [55, 56] | Houston, Texas, USA | RCT | Int N = 136  Ctrl N = 119 | Int = 4.7±0.5  Ctrl = 4.4±0.5 | Int = 44.8  Ctrl = 55.4 | **Program Name**: Strategies for Effective Eating Development (SEEDS)  **Theory**: Dialogue approach, self-determination theory, logic model.  **Channels delivered**: Bilingual staff members with a degree in psychology and/or education  **Activities:** Lessons, videos, and experiential learning activities about eating, self-regulation, and healthy preferences; discussion-based format of the lessons. | 7 weeks; weekly sessions | Y | **Level**: Family  **Setting**: Community/Clinic | **BMI:** N  **BMIz:**  Height (Seca) and weight-measuring scale (Health o Meter)  **BMI percentile:** CDC Growth Charts  **WC:** N  **Body Fat %:** N |
| Johnston et al., 2011 [57] | Houston, Texas, USA | RCT | Int N = 212  Ctrl N = 145 | 12.0±0.7 | 45 | **Program Name:** ^a^NR  **Theory:** ^a^NR  **Channels delivered:** Undergraduate students trained in physical activity/nutrition, and a bachelor-level instructor trained in nutrition  **Activities:** Nutrition instruction and physical activity training; monthly meetings with parents to teach them how to adapt family meals  and activities to facilitate healthy changes. | 6 months;  Nutrition was once/week and physical activity was twice/week for 40 min | Y | **Level:** Individual, Family  **Setting**: School | **BMI:** N  **BMIz:** Height and weight-measuring scale (standardized by age/sex)  **BMI percentile:** N  **WC:** N  **Body Fat %:** N |
| Kong et al., 2010 [58] | Albuquerque, New Mexico, USA | Quasi w/out Control | N = 25 | 5-11 | 64 | **Program Name:** Walking School Bus (WSB)  **Theory:** ^a^NR  **Channels delivered:** Physicians, premedical and medical students, and parent volunteers.  **Activities:** Participants walked on designated routes with pick-up and drop-off locations. Health-related themes were emphasized during the walks: (a) get up and play, (b) turn off your television, (c) eat 5 servings of fruit/vegetables per day, and (d) reduce soda/juice intake. | 2.5 months | Y | **Level**: Individual, Family  **Setting**: School, Community | **BMI/BMIz:** ^a^NR  **BMI percentile:** ^a^NR  **WC:** N  **Body Fat %:** N |
| Laviolette et al., 2023 [59] | Rio Grande Valley, Texas, USA | Quasi w/out Control | N = 57 | 10.21±0.9 | 52.6 | **Program Name: *¡****Haz Espacio para Papi!*  **Theory:** Family-Centered Action Model of Intervention Layout and Implementation Approach, Family Ecological Model, Family Systems Theory, and Social Cognitive Theory  **Channels delivered:** Nutrition, behavioral, social health, and physical activity instructors, a licensed family psychologist, and *promotoras*  **Activities:** Three intervention components including “mini” nutrition education lessons with food tastings, interactive lessons for participants to gain knowledge and skills related to nutrition and family functioning, child-focused cooking lessons, and eating together lessons. A mixture of in-person group sessions, check-ins (home visits and phone calls), and at-home activities occurred between the group sessions. | 6 weeks;  weekly sessions | Y | **Level:** Family  **Setting:** Home | **BMI:**  Height and weight-measuring scale (CDC BMI-for-Age Growth Charts)  **BMIz:** ^a^NR  **BMI percentile:** CDC Growth Charts  **WC:** N  **Body Fat %:** N |
| Lee et al., 2012 [60] | Laredo, Texas, USA | RCT | Int N = 10  Ctrl N = 10 | 10.4±1.2 | 0 | **Program Name:** Active Intervention Program  **Theory**: ^a^NR  **Channels delivered**: Nutritionists and personal trainers  **Activities:** Four dietary education sessions, dietary log, pedometer log, and goal setting, structured exercise sessions. | 2.5 months  Three sessions per week | Y | **Level**: Individual  **Setting**: School | **BMI:** ^a^NR  **BMIz:** N  **BMI percentile:** N  **WC:** Flexible Tape  **Body Fat %:**  Skin fold caliper (Beta Technology) |
| Lin et al., 2023 [61] | Imperial County, California, USA | Quasi w/ Control | Int N = 917  Ctrl N = 272 | 6.4±2.7 | 50.5 | **Program Name:** California Childhood Obesity Research Demonstration (CA-CORD)  **Theory:** Social Cognitive Theory  **Channel delivered:** Health care providers and community health workers  **Activities:** Three dietary interventions were conducted across various community settings, including Federally Qualified Health Centers, elementary schools, recreation centers, and restaurants. | 12 months  **School/Daycare:** Daily  **Workshops:** Bi-weekly or Monthly  **Newsletters:** Monthly (12 total)  **Coaching Calls:** Periodically (4 total)  **Clinical Counseling:** As needed | Y | **Level:** Family, Community, Environmental  **Setting:** School, Community, Clinic | **BMI:** ^a^NR  **BMIz:** N  **BMI percentile:** N  **WC:** Flexible Tape  **Body Fat %:**  Skin fold caliper (Beta Technology) |
| Lu et al., 2018 [62] | Santa Ana, California, USA | Quasi w/out Control | N = 19 | 9 | 32 | **Program Name:** ^a^NR  **Theory**: ^a^NR  **Channels delivered**: Physical education specialists and undergraduate student volunteers  **Activities:** Aerobic age-appropriate activities/games (e.g., soccer, adapted ball games), and a small component of muscle strength activities (e.g., band exercises, push-ups, & pull-ups). | 4 months; 3 weekly 45-min sessions | N | **Level**: Individual  **Setting**: School | **BMI:** Height and weight-measuring scale  **BMIz:** N  **BMI percentile:** CDC Growth Charts  **WC:** N  **Body Fat %:** N |
| Moreno et al., 2015 [63] | Houston, Texas, USA | Quasi w/out Control | n = 257 | 12.0 ± 0.6 | 52% | **Program Name:** Family Lifestyle Overweight (FLOW) Prevention Program  **Theory**: ^a^NR  **Channels delivered:** Research team, school staff, college undergraduate assistants  **Activities:** Nutrition and physical activity health education; students were provided with a daily afternoon snack of peanuts or peanut butter, intended to improve snacking and eating habits. | 6 months | Y | **Level**: Individual  **Setting**: School | **BMI/BMIz:** Height and weight-measuring scale  (standardized by age/sex)  **BMI percentile:** N  **WC:** N  **Body Fat %:**  Skin fold calipers |
| Nicklas et al., 2013 [64] | Houston, Texas, USA | RCT | Int N = 151  Ctrl N = 144 | 4.3±0.5 | 45.7 | **Program Name:** Children in Action Program (CIA)  **Theory**: Social Cognitive Theory  **Channels delivered:** Teachers, aides, and research staff facilitators  **Activities:** Physical activity SPARK lessons, introduction of basic motor skill development (e.g., object manipulation). | 5 months; weekly sessions | N | **Level**: Individual  **Setting**: School | **BMI:** N  **BMIz:**  Height and weight-measuring scale  **BMI percentile:** CDC Growth Charts  **WC:** N  **Body Fat %:** N |
| Norman et al., 2016 [65] | San Diego, California, USA | RCT | Int N = 53  Ctrl N = 53 | 11.9±0.9 | 50.9 | **Program Name:** ^a^NR  **Theory:** Chronic Care Model and Social Cognitive Theory  **Channels delivered:** Primary care providers  **Activities**: Brief counseling on healthy dietary and physical activity behaviors; health educator home visits discussing weight management, identifying barriers, and brainstorming problem-solving strategies. | 12 months; monthly mailed materials | Y | **Level**: Individual  **Setting**: Clinic | **BMI/BMIz:** Height and weight-measuring scale (standardized by age/sex)  **BMI percentile:** CDC Growth Charts  **WC:** ^a^NR  **Body Fat:** Dual-energy X-ray Absorptiometry (Hologic) |
| O'Connor, et al., 2011 [66] | Houston, Texas, USA | RCT | Int N = 20  Ctrl N = 20 | Int = 7.0±1.0  Ctrl = 6.6±1.1 | 80 | **Program Name:** Healthy Activity and Nutrition Directions (Helping HAND)  **Theory**: Social Cognitive Theory and Parenting Theory  **Channels delivered:** Children’s Health Plan health promotion specialists  **Activities**: Family sessions where goals were made for behaviors (e.g., eat more fruit/vegetables); parents completed a goal-setting worksheet with children. | 6 months; monthly sessions | Y | **Level**: Family  **Setting**: Clinic | **BMI:** N  **BMIz:** Height (Seca) and weight-measuring scale (Health-o-meter) (standardized by age/sex)  **BMI percentile:** CDC Growth Charts  **WC:** N  **Body Fat:** N |
| Ofili, 2013 [67] | Long Beach, California, USA | Quasi w/out Control | N = 19 | 10-11 | 0 | **Program Name:** ^a^NR  **Theory:** ^a^NR  **Channels delivered:** Principal investigator  **Activities:** Nutrition education  class during recess; used the ChooseMyPlate food guide for the health education component. | 3 months; weekly 10-min sessions | N | **Level**: Individual  **Setting**: School | **BMI:** Height measured with manual measuring tape, weight measured with scale  **BMIz:** N  **BMI percentile:** CDC Growth Charts  **WC:** N  **Body Fat:** N |
| Olvera et. al., 2013 [68] | Houston, Texas, USA | Quasi w/out Control | N = 61 | 10.9±1.6 | 100 | **Program Name:** BOUNCE Behavior Opportunities Uniting in Nutrition, Counseling, and Exercise (BOUNCE)  **Theory:** The Social Cognitive Theory  **Channels delivered:** Exercise sessions led by instructors certified by the Cooper Institute  **Activities:** Group sessions of physical activity with varied intensities, including lunch, a nutrition lesson, and a behavioral counseling session | 4 months; daily sessions for 3 months followed by weekly sessions | Y | **Level**: Individual  **Setting**: Community center | **BMI:** Height (Seca) and weight-measuring scale (Tanita)  **BMIz:** N  **BMI percentile:** CDC Growth Charts  **WC:** Flexible Tape  **Body Fat:**  Biolectrical impedance (Tanita) |
| Olvera et al., 2023 [69] | Houston, Texas, USA | RCT | Int N = 80 Ctrl N = 68 | 10.1±1.3 | 52 | **Program Name:** Lace Up and Mindfulness (LUAM)  **Theory:** ^a^NR  **Channels delivered:** Certified fitness instructors  **Activities:** Physical activity (i.e., kickboxing, spinning/cycling, and fitness yoga); Mindful practices (i.e., breathing exercises, meditation, body awareness); and Nutrition sessions (i.e., mindful eating, healthy snack demonstrations). | 3 months; bi-weekly 60-min sessions | N | **Level:** Individual  **Setting:** School | **BMI:** Height (Seca) and weight-measuring scale (Tanita)  **BMIz:** N  **BMI percentile:** CDC Growth Charts  **WC:** N  **Body Fat:**  Biolectrical impedance (Tanita) |
| Parra-Medina et al., 2015 [70] | San Antonio, Texas, USA | Quasi w/out Control | N = 72 | 12 (10–14) | 49.1 | **Program Name:** Y Living Program  **Theory:** Social Cognitive Theory  **Channels delivered:** YMCA staff or community volunteers (e.g., registered dietitians)  **Activities:** Health education sessions on physical activity and nutrition topics (e.g., MyPlate messages, nutrition labels, and types of exercise), wellness consultation, and special events (e.g., grocery store tours and community events). | 3 months; twice-weekly sessions | Y | **Level:** Family  **Setting**: Community center | **BMI:** N  **BMIz:** Height and weight-measuring scale (Tanita) (standardized by age/sex)  **BMI percentile:** CDC Growth Charts  **WC:** Flexible Tape (MyoTape)  **Body Fat %:** Y |
| Parsons et al., 2019 [71] | Avalon, California, USA | Quasi w/ Control | Int N = 11  Ctrl N = 11 | Int = 12±1.4  Ctrl = 11.5±1.2 | 100 | **Program Name:** We Can! Energize our Families  **Theory**: Social Cognitive Theory, Bronfenbrenner's Ecological Systems Theory Model  **Channels delivered:** Board-certified family nurse practitioner  **Activities:** Health education sessions to increase parental knowledge related to healthy meal planning (e.g., portion sizes, healthy eating) and physical activity (duration, intensity, and types). | 1 month; weekly sessions | Y | **Level**: Individual, Family  **Setting**: School | **BMI:** Height and weight-measuring scale  **BMIz:** N  **BMI percentile:** CDC Growth Charts  **WC:** N  **Body Fat %:** N |
| Pérez et al., 2024 [72] | South Los Angeles, California, USA | Cluster RCT | Int N = 18  Ctrl N = 32 | 10.9±3.6 | 48 | **Program Name:** Eat, Pray, Move  **Theory:** Socioecological Model  **Channels delivered:** Bilingual staff  **Activities:** Faith-based multilevel obesity intervention aimed to support healthy eating and increase physical activity through multilevel strategies including: daily mobile messages and educational handouts, cooking/nutrition and PA classes, sermons about nutrition and PA, a church garden; and mapping of the community food and PA environments. | 4-5 Months | Y | **Level:** Individual, Interpersonal, Organizational, and Community  **Setting:** Churches | **BMI:** Height and weight-measuring scale  **BMIz:** N  **BMI percentile:** CDC Growth Charts  **WC:** N  **Body Fat %:** N |
| Quinones et al., 2025 [73] | New Mexico, USA | Quasi w/out Control | N = 39 | 10.6±4.3 | 38.5 | **Program Name:** ^a^NR  **Theory:** Orem’s Self-Care Deficit Theory and Johns Hopkins Evidence-Based Practice Model  **Channels delivered:** Nurses and professional health providers  **Activities:** Nurse-led visits and education facilitated the patient and family in identifying a physical activity intervention that was suitable for them. | 12 weeks | Y | **Level:** Community  **Setting:** Community-Based Pediatric Clinic | **BMI:** Height and weight-measuring scale  **BMIz:** N  **BMI percentile:** N  **WC:** N  **Body Fat %:** N |
| Ramírez-López et al., 2005 [74] | Hermosillo, Sonora, México | Quasi w/ Control | Int N = 106  Ctrl N = 254 | Int = 8.6±1.3  Ctrl = 8.4±1.3 | ^a^NR | **Program Name:** *Programa de Desayunos Escolares* [PDE]  **Theory:** ^a^NR  **Channels delivered:** School committee member  **Activities:** A school committee member distributed breakfast to students in the intervention group 30 minutes before the start of class. | 9 months | Y | **Level:**  Individual,  Interpersonal,  Community  **Setting:** Public school | **BMI:** Height (Holtain) and weight-measuring scale (AND FV-150)  **BMIz:** CDC Growth Charts  **BMI percentile:** N  **WC:** N  **Body Fat %:**  Biolectrical impedance (Impedimed) |
| Ramírez-Rivera et al., 2021 [75] | Hermosillo,Sonora, México | RCT | Int N = 21  Ctrl N = 20 | 10.2±0.5 | 48.7 | **Program Name:** Planet Nutrition  **Theory:** ^a^NR  **Channels delivered:** Research team  **Activities:** Children in the intervention group received nutrition education (e.g., reading food labels, ultra-processed food) and physical activity sessions. Parents were sent six brochures with nutrition and health topics and a booklet with ideas for healthy snack preparation. | 9 weeks; bi-weekly 60-min nutrition sessions & three/week 60-min physical activity sessions | Y | **Level:** Individual, interpersonal, community  **Setting:** School and Home | **BMI:** N  **BMIz:** Height (Seca) and weight-measuring scale (Seca)  **BMI percentile:** N  **WC:** Flexible Tape  **Body Fat %:**  Biolectrical impedance (RJL Quantum II) |
| Ramírez-Rivera et al,, 2025  [76] | Hermosillo, Sonora, México | Pilot RCT | Int N = 27  Ctrl N = 27 | 10.1±0.8 | 65 | **Program Name:** Planet Nutrition (Pilot) Program  **Theory:** ^a^NR  **Channels delivered:** Nutritionist, trained nutrition interns, physical activity teachers  **Activities:** Children received nutrition education and physical activity (behavior change focus: increasing fruits and vegetable intake, reducing screen time, etc), parents were part of a private Facebook group where health and nutrition information was uploaded 2 times per week. | 4 months;  60-min sessions three times a week | Y | **Level:** Family  **Setting:** Home | **BMI:** Height (Seca) and weight-measuring scale (Tanita)  **BMIz:** WHO Anthro Plus software  **BMI percentile:** N  **WC:** Flexible Tape (Lufkin Executive Thinline)  **Body Fat %:** N |
| Ranjit et al., 2022 [77] | Dove Spring, Texas, USA | RCT | Int N = 150  Ctrl N = 163 | 31.5 | 91 | **Program Name:** Go! Austin/Vamos! Austin (GAVA)  **Theory:** ^a^NR  **Channels delivered:** Academic staff and community organizations  **Activities:** Intervention targeting the built and social environments of PA and nutrition. | 4 years | Y | **Level:** Community  **Setting:** School, Community | **BMI:** Height and weight-measuring scale  **BMIz:** N  **BMI percentile:** N  **WC:** N  **Body Fat %:** N |
| Rehkopf et al., 2023 [78] | Stanford, California, USA | Quasi w/out Control | N = 130 | 10.5±1.4 | 55 | **Program Name:** CHANGES  **Theory:** ^a^NR  **Channels delivered:** ^a^NR  **Activities:** Sessions on improving dietary energy density, physical activity reducing screen time, and improving parenting practice to reduce weight gain. | 6 months | Y | **Level:** Individual, Family  **Setting:** ^a^NR | **BMI:** ^a^NR  **BMIz:** N  **BMI percentile:** CDC Growth Charts  **WC:** ^a^NR  **Body Fat %:** N |
| Rhee et al., 2022 [79] | San Diego County, California, USA | RCT | Int N = 82  Ctrl N = 82 | 9.6 | 49 | **Program Name:** Guided Self-Help (GSH)  **Theory:** Self-Regulation Theory  **Channels delivered:** Bilingual, bicultural health coaches  **Activities:** Received standard behavioral strategies. Parent and child received GSH materials (i.e., problem-solving, stimulus control, self-monitoring, and goal setting). | 6 months; first 4 sessions were weekly, next 10 sessions biweekly  Session 1 was one-hour, subsequent sessions were 20 minutes | Y | **Level:** Individual, Family  **Setting:** Primary Care Clinics and Schools | **BMI:** Height (Schorr) and weight-measuring scale (Tanita)  **BMIz:** CDC Growth Charts  **BMI percentile:** CDC Growth Charts  **WC:** N  **Body Fat %:** N |
| Ríos-Reyna et al., 2022 [80] | Reynosa, Tamaulipas, México | Quasi w/ Control | Int N = 167  Ctrl N = 142 | 9.7±0.5 | 52.3 | **Program Name:** ^a^NR  **Theory:** ^a^NR  **Channels delivered:** ^a^NR  **Activities:** Children received nutrition education and flyers/materials were sent home to their parents | 7 weeks; 20-min weekly sessions | Y | **Level:** Individual, Family  **Setting:** School | **BMI/BMIz:** Height (Seca) and weight-measuring scale (Tanita)  **BMI percentile:** N  **WC:** N  **Body Fat %:** N |
| Ríos-Reyna et al., 2025 [81] | Reynosa, Tamaulipas, México | RCT | Int N = 297  Ctrl N = 338 | 10.2±0.9 | 51.5 | **Program Name:** ^a^NR  **Theory:** Social Cognitive Theory **Channels delivered:** Nutrition graduates and trained psychology students  **Activities:** Received health education on nutrition, emotion management, and body image perceptions. Brochures were sent to parents, and posters were displayed at school | 14 weeks; 30-mi weekly sessions | Y | **Level:** Individual, Family  **Setting:** School | **BMI/BMIz:** Height (Seca) and weight-measuring scale (Tanita)  **BMI percentile:** N  **WC:** N  **Body Fat %:** N |
| Robinson et al., 2021 [82] | Stanford, California, USA | RCT | Int N = 120  Ctrl N = 118 | Int **=**  9.5±1.4  Ctrl = 9.5±1.5 | Int **=** 58  Ctrl = 54 | **Program Name:** Stanford GOALS  **Theory:** Behavioral change theory; Social cognitive model  **Channels delivered:** Trained bilingual health educators in Spanish and English  **Activities:** GOALS was an after-school sports program where children engaged in 4 sports (e.g., track and field, volleyball, rugby, and swimming). Children also received five modules that included various topics (e.g., home environment, promotion of eating, physical activity, and screen time). | 3 years | Y | **Level:** Individual, Interpersonal, Community  **Setting:** School and Home | **BMI:** Height and weight-measuring scale  **BMIz:** N  **BMI percentile:** N  **WC:** Flexible Tape  **Body Fat %:** ^a^NR |
| Robles et al., 2014 [83] | Bailey County, Texas, USA | Quasi w/out Control | N = 31 | 9.6±0.8 | 81 | **Program Name:** ^a^NR  **Theory:** ^a^NR  **Channels delivered:** Pharmacists, pharmacy student researcher, and junior high school coach  **Activities:** Alternating health education between nutrition and physical activity topics, such as dietary guidelines, reducing intake of sugary foods, portion sizes, reducing sedentary behavior, and a study-hosted track meet. | 1 month; twice-weekly 60 min sessions | N | **Level:** Community  **Setting:** Community center and park | **BMI:** Height and weight-measuring scale  **BMIz:** N  **BMI percentile:** ^a^NR  **WC:** Flexible Tape  **Body Fat %:** N |
| Romero et al., 2014 [84] | Sonora, Mexico | Quasi w/ Control | Int N = 59  Ctrl N = 60 | 8-11 | 59.7 | **Program Name:** ^a^NR  **Theory:** ^a^NR  **Channels delivered:** ^a^NR  **Activities:** Physical activity sessions had three parts: (1) warm-up; (2) strength, endurance, flexibility, and coordination exercises; (3) relaxation and stretching exercises. | 5 months; twice-weekly 60 min sessions | N | **Level:**  Individual,  Community  **Setting:** Public school | **BMI/BMIz:** Height (Holtain) and weight-measuring scale (Seca)  **BMI percentile:** Organización Mundial de la Salud 2010 Classifications  **WC:** N  **Body Fat %:** N |
| Romero-Pérez, Enríquez, et al., 2020 [85] | Hermosillo,Sonora, Mexico | RCT | Int N = 48  Ctrl N = 47 | 10.3±0.8 | ^a^NR | **Program Name:** ^a^NR  **Theory:** ^a^NR  **Channels delivered:** First author and researcher  **Activities:** Sessions varied with aerobics, strength, conditioning, and coordination exercises. Sessions were in addition to the regular PE classes. | 5 months; twice-weekly 60-min sessions | N | **Level:** Individual, Community  **Setting:** School | **BMI:** Height (Seca) and weight-measuring scale (Seca)  **BMIz:** World Health Organization software  **BMI percentile:** N  **WC:** N  **Body Fat %:** N |
| Romero-Pérez, González-Bernal et al., 2020 [86] | Hermosillo,Sonora, Mexico | Quasi w/ Control | Int N = 54  Ctrl N = 51 | 10.0±0.8 | 57.1 | **Program Name:** ^a^NR  **Theory:** ^a^NR  **Channels delivered:** ^a^NR  **Activities:** Physical activity program focused on the development of conditional and coordinative capacities. Participants completed core exercises, aerobic exercises, and games. | 5 months; twice-weekly 50-min sessions | N | **Level:** Individual  **Setting:** School | **BMI:** N  **BMIz:** Height (Holtain) and weight-measuring scale (Seca)  **BMI percentile:** N  **WC:** N  **Body Fat %:** N |
| Sadeghi, Kaiser et al., 2019 [87] | Central Valley, California, USA | Quasi w/ Control | Int N = 430  Ctrl N = 352 | 6.0±1.3 | 50.7 | **Program Name:** *Niños Sanos, Familia Sana*  **Theory:** Social Learning Theory  **Channels delivered:** University of California Cooperative Extension (UCCE) nutrition educators, classroom teachers, and a PE teacher  **Activities:** Parents participated with children in monthly discussions, hands-on activities, and food demonstrations. Health education included healthy snacks, eating more fruits/vegetables, and increasing active play. | 3 years; monthly health education sessions & weekly physical activity sessions | Y | **Level:** Individual,  Interpersonal,  Community  **Setting:** School | **BMI/BMIz:** Height (Seca) and weight-measuring scale (Seca)  **BMI percentile:** N  **WC:** Y  **Body Fat %:** N |
| Salvo et al., 2019 [88] | Austin, Texas, USA | Quasi w/out Control | N = 286 | ^a^NR | 47.6 | **Program Name:** Go Austin! *¡Vamos, Austin!* Evaluation (GAVA) Evaluation Study  **Theory:** Socio-Ecological Model  **Channels delivered:** Organizational partners from the community  **Activities:** Organizational partners received capacity-building support and training to improve their resources, making them more accessible to residents. | 5 years | Y | **Level:** Community  **Setting:** Community | **BMI:** Height and weight-measuring scale  **BMIz:** N  **BMI percentile:** CDC Growth Charts  **WC:** N  **Body Fat %:** N |
| Shaibi et al., 2010 [89] | Phoenix, Arizona, USA | Quasi w/out Control | N =102 | 11.8±3.0 | ^a^NR | **Program Name:** Every Little Step Counts (ELSC)  **Theory:** ^a^NR  **Channels delivered:** Bilingual and bicultural registered dieticians and promotoras de salud (community health workers)  **Activities:** Parents and children attended health education sessions discussing diabetes, nutrition education, physical activity, roles and responsibilities of communication and health, self-esteem and self-efficacy discussions, and personal reflections of lifestyle goals. Families also had one-on-one life management appointments with the dietician 12 weeks after they attended a class for problem-solving and counseling. | ^a^NR | Y | **Level:** Individual,  Interpersonal,  Community  **Setting:** ^a^NR | **BMI:** ^a^NR  **BMIz:** N  **BMI percentile:** N  **WC:** N  **Body Fat %:** N |
| Sharma et al., 2019 [90] | Houston, Texas, USA | RCT | Int N = 483  Ctrl N = 365 | Int = 4.1±0.7  Ctrl = 4.2±0.7 | Int = 48.2  Ctrl = 47.4 | **Program Name:** Coordinated Approach to Child Health Early Childhood (CATCH EC)  **Theory:** Social Cognitive Theory  **Channels delivered:** Preschool staff (directors, teachers, and parents)  **Activities:** Preschool staff received 4-6 hours of training on the CATCH EC curriculum. Children learn about nutrition, gardening, and indoor and outdoor physical activity. Parents receive tip sheets that include recipes, meal plans, parent-child activities, diet recommendations for preschool children, physical activity, and screen time. | 2 years; weekly 60-min sessions | Y | **Level:** Individual,  Interpersonal,  Community  **Setting:** School | **BMI/BMIz:** Height (Perspective Enterprises) and weight-measuring scale (Tanita)  **BMI percentile:** CDC Growth Charts  **WC:** N  **Body Fat %:** N |
| Shinn et al., 2017 [91] | Santa Ana, California, USA | Quasi w/out Control | N = 43 | 7.0±2.7 | 58 | **Program Name:** Family Mealtime Coaching (FMC)  **Theory:** Positive Psychology (theory/model not specified)  **Channels delivered:** Coaches such as psychologists, social workers, marriage and family counselors, or any professional with a master’s or higher in a mental health field  **Activities:** Coaches received a 5-hour training. Parents in the FMC group received live coaching while they prepared a meal. A clinician then coached parents on decreasing maladaptive feeding strategies while they ate the meal prepared. The parent-child dyads also participated in vigorous play. | 8 one-hour sessions | Y | **Level:** Individual,  Interpersonal,  Community  **Setting:** Community center and Community mental health agency (i.e., Child Guidance Center) | **BMI:** N  **BMIz:** ^a^NR  **BMI percentile:** ^a^NR  **WC:** Flexible Tape  **Body Fat %:** N |
| Singh et al., 2020 [92] | Los Angeles, California, USA | Quasi w/out Control | N = 52 | 9.3 (5-12) | 58 | **Program Name:** Healthy Eating Lifestyle Program (HELP)  **Theory:** ^a^NR  **Channels delivered:** Medical residents, medical students, registered dietitians/certified diabetes educators  **Activities:** Cooking instruction and supermarket tours, food guide to plant-based eating, PA pyramid, and pedometer. | 1.5 months; weekly sessions | Y | **Level:** Family  **Setting:** Clinic | **BMI:** N  **BMIz:** Height and weight-measuring scale; CDC Growth Charts  **BMI percentile:** N  **WC:** N  **Body Fat %:** N |
| Slusser et al., 2012 [93] | Los Angeles, California, USA | RCT | Int N = 61  Ctrl N = 60 | 2-4 | Int = 55.7  Ctrl = 56.7 | **Program Name:** Pediatric Overweight Prevention through Parent Training Program (PT)  **Theory:** ^a^NR  **Channels delivered:** Bilingual social worker and trained bilingual master's in public health educator  **Activities:** Parents completed health education modules on praising appropriate behavior, increasing dietary knowledge, giving clear commands, implementing consistent daily routines, and setting limits/warnings. | 12 months; nine 90-min sessions | Y | **Level:** Interpersonal  **Setting:** Venice Family Clinic Simms/Mann Health and Wellness Center, Los Angeles Unified School District preschools, Santa Monica Head Start Program, the Mar Vista Family Center, and the Children’s Bureau | **BMI:** Height and weight-measuring scale (Seca)  **BMIz:** N  **BMI percentile:** CDC Growth Charts  **WC:** N  **Body Fat %:** N |
| Smith et al., 2021 [94] | Maricopa, Arizona, USA | RCT | Int N = 141 Ctrl N = 99 | 9.5± 2.0 | 49 | **Program Name:** Family Check-Up 4 Health (FCU4Health)  **Theory:** Family-Centered Behavioral Framework  **Channels delivered:** Trained clinic staff  **Activities:** Parents were provided with the skills and support to implement the recommended diet, physical activity, and family health routine changes. | 6 months;  26-50 hours per week | Y | **Level:** Family  **Setting:** Clinic | **BMI:** N  **BMIz:** Height and weight-measuring scale (Tanita) (standardized by age/sex)  **BMI percentile:** ^a^NR  **WC:** ^a^NR  **Body Fat %:** Biolectrical impedance (Tanita) |
| Soltero et al., 2019 [95] | Maryvale, Arizona, USA | Quasi w/out Control | N = 68 | 9.8±1.3 | 57.1 | **Program Name:** ¡*Viva Maryvale*!  **Theory:** Social Cognitive Theory  **Channels delivered:** Bilingual/bicultural registered dieticians and community health educators for nutrition education sessions. YMCA fitness instructors for physical activity  **Activities:** Parents and children were separated for some nutrition sessions. Children also participated in physical activity sessions. One physical activity session included the entire family. | 3 months; 4 60-min sessions per week | Y | **Level:** Individual,  Interpersonal  **Setting:** Medical home | **BMI:** N  **BMIz:** Height and weight-measuring scale  **BMI percentile:** ^a^NR  **WC:** ^a^NR  **Body Fat %:** Biolectrical impedance |
| Stevens et al., 2011 [96] | Phoenix, Arizona, USA | Quasi w/out Control | N = 17 | 11-14 | 58.8 | **Program Name:** *Salud con Sabor Latino para los Niños* (SSLN)  **Theory:** Social Cognitive Theory  **Channels delivered:** Trained staff from the Golden Gate Community Center: Health Coordinator, wellness instructor, and promotoras delivered nutrition sessions. A member of the research team delivered the physical activity curriculum.  **Activities:** Health education on nutrition and physical activity (e.g., cues for hunger, setting personal goals, food labels, and Zumba). | 2 months; twice-weekly 30-min sessions | N | **Level:** Individual  **Setting:** Golden Gate Community Center | **BMI:** ^a^NR  **BMIz:** ^a^NR  **BMI percentile:** ^a^NR  **WC:** ^a^NR  **Body Fat %:** N |
| Teran-Garcia et al., 2023 [97]^c^ | Texas, Iowa, and CA | RCT | Int N = 239  Ctrl N = 187 | 10.15±2.9 | 55 | **Program Name:** *Abriendo Caminos*  **Theory:** Social Cognitive Theory  **Channels delivered:** Trained health, nutrition, food science, child and family development, and kinesiology students, Cooperative Extension educators, community partners, and research staff also assisted.  **Activities:** Workshop sessions included three components: nutrition, family wellness, and physical activity**.** | 6 weeks;  2-hour sessions | Y | **Level:** Family  **Setting:** Home, Community | **BMI:** N  **BMIz:** Height and weight-measuring scale (Seca) (standardized by age/sex)  **BMI percentile:** CDC Growth Charts  **WC:** N  **Body Fat %:** N |
| Treviño et al., 2004 [98] | San Antonio, Texas, USA | RCT | Int N = 706  Ctrl N = 713 | Int =  9.8±0.5  Ctrl = 9.8±0.5 | Int =  49  Ctrl = 50 | **Program Name:** Bienestar Health Program  **Theory:** Social cognitive theory and social ecological theory  **Channels delivered:** Classroom teachers conducted health sessions. Parents and school food service workers encouraged healthy behaviors.  **Activities:** Sessions included topics associated with diabetes control, like decreasing saturated fat intake, increasing dietary fiber intake, and increasing physical activity. Students also participated in a health club. Parents received a workbook that included cooking demonstrations, salsa dancing, a nutrition-related loteria bingo game, and a physical activity called the wheel of health game. School food service workers were given a workbook to improve nutrition knowledge. | 7 months  **Health and physical activity sessions:** 45 minutes a day, 5 days a week. One day is health education and 4 days are physical activity.  **Health club:** Once a week after school for 1 hour.  **Parents:** Monthly meetings every other month.  **School food service**: One lesson per month, each lesson 30 min. | Y | **Level:** Interpersonal,  Intrapersonal,  Community  **Setting:** Elementary schools | **BMI:** Height and weight-measuring scale (Seca) (Quetelet Index Measure)  **BMIz:** N  **BMI percentile:** N  **WC:** N  **Body Fat %:** Biolectrical impedance (Tanita) |
| Trinidad et al., 2024 [99] | Long Beach, California, USA | Quasi w/out Control | N = 56 | 10-13 | ^a^NR | **Program Name:** Eat, Play, Go!  **Theory:** Social Cognitive Theory  **Channels delivered:** Undergraduate and Graduate Students  **Activities:** Health education and booster sessions were conducted on nutrition knowledge, dietary patterns (i.e., intake of fruits/vegetables, sugar-sweetened beverages, & breakfast), and physical activity. | 2 months  1 month; 4 weekly sessions followed by 2 monthly booster sessions | Y | **Level:** Family  **Setting:** Community | **BMI:** Height and weight-measuring scale (Seca) (Quetelet Index Measure)  **BMIz:** N  **BMI percentile:** N  **WC:** N  **Body Fat %:** Biolectrical impedance (Tanita) |
| Tucker et al., 2023 [100] | Santa Cruz County, Arizona, USA | Quasi w/ Control | Int N = 83  Ctrl N = 81 | 7.2±1.2 | 23-25 | **Program Name:** *La Vida Buena*  **Theory:** Socio-Ecological Model  **Channels delivered:** *Promotoras*  **Activities:** Health education sessions covered nutrition, physical activity, and parenting strategies. Physical activity interactive sessions were conducted where children and parents engaged in moderate-to-vigorous physical activities together. | 8 weeks; 60-min weekly session | Y | **Level:** Individual, Family, Community  **Setting:** Community | **BMI/BMIz:** Height and weight-measuring scale  **BMI percentile:** N  **WC:** N  **Body Fat %:** N |
| VanEvery et al., 2023 [101] | San Diego, California, USA | RCT | Low N = 28  High N = 30 | 9.4±2.2 | 12-16 | **Program Name:** ^a^NR  **Theory:** ^a^NR  **Channels delivered:** Dietitian-trained community health workers  **Activities:** Families received nutrition education throughout the 6-month period, which provided families with tools to improve diet quality and meet nutritional goals. | 6 months;  12 bi-weekly sessions | Y | **Level:** Family  **Setting:** Clinic | **BMI:** Height and weight-measuring scale  **BMIz:** N  **BMI percentile:** N  **WC:** Semi-flexible Tape  **Body Fat %:** N |
| Webb et al., 2025  and Farbo, 2022 [102, 103]*^b^* | Texas, USA | Quasi w/ Control | Int N = 59 Ctrl N = 49 | 7-8 | 52.7 | **Program Name:** Let's inspire innovation ‘N Kids **(**LiiNK) Project  **Theory:** Positive Action  **Channels delivered:** Teachers  **Activities:** Physical education classes to improve muscular strength | 1 school year;  daily sessions | N | **Level:** Individual  **Setting:** School | **BMI:** N  **BMIz:** N  **BMI percentile:** N  **WC:** N  **Body Fat %:**  Biolectrical impedance |
| Wiersma and Rubin, 2012 [104] | Fullerton, California, USA | Quasi w/out Control | N = 18 | Girls N =  9±1.7  Boys N =  7.5±1.7 | 61.1 | **Program Name:** Active Kids  **Theory:** ^a^NR  **Channels delivered:** Research team  **Activities:** Children participated in games-based physical activity focusing on moderate to vigorous physical activity (e.g., tag, obstacle course). | 15 weeks; twice-weekly 60-min sessions | N | **Level:** Individual  **Setting:** Local park | **BMI:** Height and weight-measuring scale (Detecto 349)  **BMIz:** N  **BMI percentile:** CDC Growth Charts  **WC:** Flexible Tape (Gulik)  **Body Fat %:**  Skin fold calipers (Beta Technology) |
| Winter et al., 2011 [105] | San Antonio, Texas, USA | Quasi w/ Control | Int N = 206  Ctrl N = 194 | 4.2 ± 0.6 | 91 | **Program Name:** Healthy and Ready to Learn  **Theory:** Social Cognitive Theory  **Channels delivered:** Teachers, parents, and *promotoras*  **Activities:** Children received bilingual (English and Spanish) activity packets with six modules focused on improving school readiness and health strategies for preventing obesity (nutrition and physical activity).  Parent sessions showed them how to implement child activities at home. Teachers engaged in 20 hours of training, which covered obesity prevention topics. | 6 months | Y | **Level:** Individual,  Interpersonal,  Community  **Setting:** Home and School | **BMI:** Height and weight-measuring scale (Tanita)  **BMIz:** N  **BMI percentile:** N  **WC:** N  **Body Fat %:** N |
| Wong et al., 2016 [106] | Houston, Texas, USA | Quasi w/ Control | Int N = 524  Ctrl N = 353 | 10.3±.04 | Int = 50.6  Ctrl = 44.2 | **Program Name:** Healthy Kids-Houston  **Theory:** ^a^NR  **Channels delivered:** Program staff trained in physical activity curricula (supervised by City of Houston Parks and Recreation Department, HPARD). Nutrition lessons supervised by a doctoral nutritional epidemiologist. Healthy habits lessons were supervised by a doctoral clinical pediatric psychologist.  **Activities:** Children in the intervention participated in physical activity, nutrition, and healthy habits lessons. | 4.5 months; twice-weekly 120 min sessions | N | **Level:** Individual,  Community  **Setting:** Head Start | **BMI/BMIz:** Height (Accustat) and weight-measuring scale (Tronix)  **BMI percentile:** ^a^NR  **WC:** N  **Body Fat %:** N |
| Wright et al., 2012 [107] | Los Angeles, California, USA | RCT | Int N = 121  Ctrl N = 130 | Int =  9±1.6  Ctrl = 8.3±1.1 | Int = 58  Ctrl = 62 | **Program Name:** Kids Nutrition and Fitness  **Theory:** Community-academic partnered participatory research (CPPR)  **Channels delivered:** Advanced practice nurse, registered nurses, a physical education specialist, and community health workers.  **Activities:** Parents and children attended the after-school Kids Nutrition and Fitness (KNF) program. They also had access to physical and mental health services in local community clinics. A School Health Advisory Council was established to support and promote school wellness policies related to nutrition and physical activity. Health promotion seminars were offered to staff, while parents received bi-monthly educational newsletters. | 1.5 months; weekly 90-min sessions | Y | **Level:** Interpersonal,  Intrapersonal,  Community,  Policy  **Setting:** Home,  School | **BMI:** Height (Harpenden) and weight-measuring scale (Detecto)  **BMIz:** Epi Info software  **BMI percentile:** N  **WC:** N  **Body Fat %:** N |
| Yin et al., 2012 [108] | San Antonio, Texas, USA | Quasi w/ Control | Int N = 66  Ctrl N = 69 | 4.1±0.6 | 55 | **Program Name:** *Míranos*! Look at Us, We Are Healthy!  **Theory:** Socioecological Model, Social developmental theory, and Social Learning Theory  **Channels delivered:** Head Start teachers and staff, research assistants, and peer parent educators  **Activities:** Center-based– Head Start teachers and staff were trained on implementing a gross-motor skills program during outdoor play. Health education was implemented during classroom time using the Sesame Street Workshop Healthy Habits for Life resource kit. Home-based intervention– A combination of center-based activities described above, in addition to involving parents to increase health literacy on obesity, nutrition, and physical activity. | 4.5 months; twice-weekly sessions | Y | **Level:** Individual,  Interpersonal,  Community  **Setting:** Head Start Centers | **BMI:** N  **BMIz:** Height and weight-measuring scale  **BMI percentile:** N  **WC:** N  **Body Fat %:** N |
| Yin et al., 2023 [109]  Ullevig et al., 2023 [110] | San Antonio, Texas, USA | RCT | Int N = 100  Ctrl = 123 | 3.6±0.3 | 57 | **Program Name:** *Míranos*! Look at Us, We Are Healthy!  **Theory:** Socioecological Model, Social developmental theory, and Social Learning Theory  **Channels delivered:** Head Start teachers and staff, research assistants, and peer parent educators  **Activities:** Center-based– Head Start teachers and staff were trained on implementing a gross-motor skills program during outdoor play. Health education was implemented during classroom time using the Sesame Street Workshop Healthy Habits for Life resource kit. Home-based intervention– A combination of center-based activities described above, in addition to involving parents to increase health literacy on obesity, nutrition, and physical activity. | 8 months;  monthly sessions, 3 home visits | Y | **Level:** Family  **Setting:** Home, School | **BMI:** N  **BMIz:**  Height and weight-measuring scale (standardized by age/sex - CDC Growth Charts)  **BMI percentile:** N  **WC:** N  **Body Fat %:** N |
| Yudkin et al., 2024 [111] | Dallas, Texas, USA | Quasi w/out Control | N = 73 | 9.3±1.8 | 29 | **Program Name:** Dynamo Kids!  **Theory:** Obesity Chronic Care model  **Channels delivered:** Website  **Activities:** Technology-based, customized, self-paced, and self-directed intervention that included an online portal in both English and Spanish. Parents were able to access to learn strategies to improve specific health behaviors for their children. | 3 months;  3 times/ week for 20 minutes | Y | **Level:** Family  **Setting:** Clinic | **BMI:** N  **BMIz:** Self-reported height and weight (standardized by age/sex)  **BMI percentile:** N  **WC:** N  **Body Fat %:** N |

**Abbreviations:** ^a^NR: Not Reported, ^b^Indicates a record from the grey literature (not peer-reviewed), ^c^Study was included because more than 50% of sample from border states. USA: United States of America, MX: Mexico, Int: Intervention, Ctrl: Control, Y: Yes, N: No, BMI: Body Mass Index; WC: Waist Circumference, PA: Physical Activity.
