# Supplementary material for: Childhood obesity interventions among Latinos residing in the US–Mexico border: a systematic review and meta-analysis of obesity outcomes
Source: Ann Behav Med. 2026 Sep 16;60(1):kaag056. doi: 10.1093/abm/kaag056 (PMC13581397; doi:10.1093/abm/kaag056)
Supplement: Supplementary file 1 [file kaag056_supplementary_data.zip › XXXXXSupplementary_data.zip!/Supplemental Material 3Updated Search_FINAL.docx]

**Supplemental Material.** Results of Subgroup and Meta-Regression Analyses in Randomized Controlled Trials Stratified by Obesity Measure

| **Group and Outcome** | **Cohen’s d** | **SE** | **96% CI** | **I^2^** | **p-value** |
| --- | --- | --- | --- | --- | --- |
| **BMI (n=17)** |  |  |  |  |  |
| Border Region |  |  |  |  | 0.87 |
| Yes | 0.07 | 0.08 | -0.08, 0.22 | 0 | 0.34 |
| No | 0.09 | 0.06 | -0.03, 0.20 | 7 | 0.13 |
| Intervention Target Level |  |  |  |  | 0.26 |
| Individual | 0.11 | 0.05 | 0.03, 0.22 | 0 | 0.03 |
| Multilevel | 0.00 | 0.09 | -0.17, 0.17 | 0 | 0.99 |
| Duration | 0.00 | 0.00 | -0.01, 0.01 | 0 | 0.87 |
| **BMIz (n=24)** |  |  |  |  |  |
| Parent Involvement |  |  |  |  | 0.47 |
| No | 0.01 | 0.01 | -0.01, 0.03 | 0.1 | 0.40 |
| Yes | -0.02 | 0.03 | -0.08, 0.05 | 0 | 0.63 |
| Intervention Target Level |  |  |  |  | 0.22 |
| Individual | 0.01 | 0.01 | -0.01, 0.03 | 0 | 0.31 |
| Multilevel | -0.04 | 0.04 | -0.11, 0.04 | 0 | 0.32 |
| Duration | 0.00 | 0.00 | -0.00, 0.00 | 0 | 0.81 |
| **BMI Percentile (n=10)** |  |  |  |  |  |
| Duration | 0.00 | 0.00 | -0.01, 0.01 | 16 | 0.75 |
| **Waist Circumference (n=12)** |  |  |  |  |  |
| Intervention Target Level |  |  |  |  | 0.30 |
| Individual | -0.02 | 0.01 | -0.04, 0.01 | 0 | 0.14 |
| Multilevel | -0.11 | 0.09 | -0.29, 0.07 | 41 | 0.23 |
| Duration | 0.01 | 0.04 | -0.08, 0.09 | 30 | 0.86 |
| **Percent Body Fat (n=10)** |  |  |  |  |  |
| Parent Involvement |  |  |  |  | 0.53 |
| No | -0.21 | 0.15 | -0.51, 0.09 | 94 | 0.18 |
| Yes | -0.10 | 0.07 | -0.23, 0.03 | 28 | 0.13 |
| Intervention Target Level |  |  |  |  | 0.49 |
| Individual | -0.19 | 0.14 | -0.47, 0.08 | 77 | 0.17 |
| Multilevel | -0.08 | 0.08 | -0.23, 0.07 | 66 | 0.29 |
| Duration | 0.03 | 0.03 | -0.04, 0.11 | 58 | 0.20 |

*Note*: Tests of subgroup differences were not reported when at least one subgroup included fewer than five studies. Bolded: Significant at p<0.05. Categorical moderators (parent involvement, border region, and intervention target level) were evaluated using random-effects subgroup analyses. Intervention duration (months) was examined using random-effects meta-regression models estimated with restricted maximum likelihood (REML).

**Supplemental Material.** Results of Subgroup and Meta-Regression Analyses in Quasi-experimental Studies with a Control Group Stratified by Obesity Measure

| **Group and Outcome** | **Cohen’s d** | **SE** | **96% CI** | **I^2^** | **p-value** |
| --- | --- | --- | --- | --- | --- |
| **BMI (n=12)** |  |  |  |  |  |
| Country |  |  |  |  | 0.14 |
| U.S. | 0.05 | 0.03 | -0.01, 0.12 | 0 | 0.12 |
| Mexico | 0.58 | 0.36 | -0.12, 1.27 | 96 | 0.11 |
| Duration | -0.00 | 0.02 | -0.04, 0.03 | 94 | 0.85 |
| **BMIz (n=11)** |  |  |  |  |  |
| Parent Involvement |  |  |  |  | **0.03** |
| No | 0.67 | 0.32 | 0.03, 1.31 | 93 | 0.04 |
| Yes | -0.01 | 0.05 | -0.12, 0.10 | 0 | 0.87 |
| Duration | 0.00 | 0.02 | -0.03, 0.04 | 93 | 0.92 |

*Note*: Tests of subgroup differences were not reported when at least one subgroup included fewer than five studies. Bolded: Significant at p<0.05. Categorical moderators (parent involvement and country) were evaluated using random-effects subgroup analyses. Intervention duration (months) was examined using random-effects meta-regression models estimated with restricted maximum likelihood (REML).

**Supplemental Material.** Results of Subgroup and Meta-Regression Analyses in Quasi-experimental Studies without a Control Group Stratified by Obesity Measure

| **Group and Outcome** | **Cohen’s d** | **SE** | **96% CI** | **I^2^** | **p-value** |
| --- | --- | --- | --- | --- | --- |
| **BMI (n=17)** |  |  |  |  |  |
| Parent Involvement |  |  |  |  | 0.47 |
| No | -0.08 | 0.08 | -0.23, 0.08 | 0 | 0.35 |
| Yes | -0.33 | 0.34 | -0.99, 0.33 | 82 | 0.33 |
| Country |  |  |  |  | 0.33 |
| U.S. | -0.01 | 0.19 | -0.40, 0.37 | 51 | 0.95 |
| Mexico | -0.35 | 0.29 | -0.91, 0.22 | 85 | 0.22 |
| Duration | 0.03 | 0.11 | -0.21, 0.27 | 83 | 0.79 |
| **BMIz (n=6)** |  |  |  |  |  |
| Duration | -0.01 | 0.14 | -0.40, 0.37 | 0 | 0.93 |
| **BMI Percentile (n=8)** |  |  |  |  |  |
| Duration | 0.003 | 0.03 | -0.08, 0.08 | 0 | 0.93 |
| **Waist Circumference (n=9)** |  |  |  |  |  |
| Duration | 0.02 | 0.08 | -0.16, 0.20 | 0 | 0.81 |
| **Percent Body Fat (n=6)** |  |  |  |  |  |
| Duration | -0.03 | 0.13 | -0.37, 0.32 | 0 | 0.85 |

*Note*: Tests of subgroup differences were not reported when at least one subgroup included fewer than five studies. Bolded: Significant at p<0.05. Categorical moderators (parent involvement, and country) were evaluated using random-effects subgroup analyses. Intervention duration (months) was examined using random-effects meta-regression models estimated with restricted maximum likelihood (REML).
